# Supplementary material for: Epidemiological and clinical features of human metapneumovirus in hospitalised paediatric patients with acute respiratory illness: a cross-sectional study in Southern China, from 2013 to 2016
Source: BMJ Open. 2018 Feb 6;8(2):e019308. doi: 10.1136/bmjopen-2017-019308 (PMC5829904; doi:10.1136/bmjopen-2017-019308)
Supplement: Author's manuscript [file bmjopen-2017-019308.draft_revisions.pdf]

BMJ Open is committed to open peer review. As part of this commitment we make the peer review history of every article we publish publicly available.

When an article is published we post the peer reviewers' comments and the authors' responses online. We also post the versions of the paper that were used during peer review. These are the versions that the peer review comments apply to.

The versions of the paper that follow are the versions that were submitted during the peer review process. They are not the versions of record or the final published versions. They should not be cited or distributed as the published version of this manuscript.

BMJ Open is an open access journal and the full, final, typeset and author-corrected version of record of the manuscript is available on our site with no access controls, subscription charges or pay-per-view fees (http://bmjopen.bmj.com).

If you have any questions on BMJ Open's open peer review process please email editorial.bmjopen@bmj.com

BMJ Open

Epidemiology and clinical features of human metapneumovirus in children hospitalized with acute respiratory tract infection from 2013 to 2016: a cross-sectional study

Journal:	*BMJ Open*
Manuscript ID	bmjopen-2017-019308
Article Type:	Research
Date Submitted by the Author:	24-Aug-2017

Complete List of Authors:	Zhang, Ling; State Key Laboratory of Respiratory Diseases, The First Affiliated Hospital of Guangzhou Medical University, Guangzhou Medical University<br>Liu, Donglan; State Key Laboratory of Respiratory Diseases, The First Affiliated Hospital of Guangzhou Medical University, Guangzhou Medical University<br>Chen, Dehui; Department of Pediatrics, The First Affiliated Hospital of Guangzhou Medical University, Guangzhou Medical University<br>Tan, Weiping ; Sun Yat-Sen Memorial Hospital, Sun Yat-Sen University<br>Qiu, Shuyan; State Key Laboratory of Respiratory Diseases, The First Affiliated Hospital of Guangzhou Medical University, Guangzhou Medical University<br>Xu, Duo; State Key Laboratory of Respiratory Diseases, The First Affiliated Hospital of Guangzhou Medical University, Guangzhou Medical University<br>Li, Xiao; State Key Laboratory of Respiratory Diseases, The First Affiliated Hospital of Guangzhou Medical University, Guangzhou Medical University<br>Liu, Tiantian; State Key Laboratory of Respiratory Diseases, The First Affiliated Hospital of Guangzhou Medical University, Guangzhou Medical University<br>Liu, Wenkuan; State Key Laboratory of Respiratory Diseases, The First Affiliated Hospital of Guangzhou Medical University, Guangzhou Medical University<br>Zhou, Rong; State Key Laboratory of Respiratory Diseases, The First Affiliated Hospital of Guangzhou Medical University, Guangzhou Medical University

**Primary Subject Heading**:	Infectious diseases
Secondary Subject Heading:	Epidemiology, Paediatrics, Infectious diseases
Keywords:	Human metapneumovirus, Epidemiology, Clinical feature, Acute respiratory illness

For peer review only

Epidemiology and clinical features of human metapneumovirus in children hospitalized with acute respiratory tract infection from 2013 to 2016: a cross-sectional study

Ling Zhang[1†], Donglan Liu[1†], Dehui Chen[2], Weiping Tan[3], Shuyan Qiu[1], Duo Xu[1], Xiao Li[1], Tiantian Liu[1], Wenkuan Liu[1]*, Rong Zhou[1]*

[1]State Key Laboratory of Respiratory Diseases, The First Affiliated Hospital of Guangzhou Medical University, Guangzhou Medical University, Guangzhou, People's Republic of China.

[2]Department of Pediatrics, The First Affiliated Hospital of Guangzhou Medical University, Guangzhou Medical University, Guangzhou, People's Republic of China.

[3]Sun Yat-Sen Memorial Hospital, Sun Yat-Sen University, Guangzhou, People's Republic of China.

*Correspondence to Professor Rong Zhou; zhourong@gird.cn and Wenkuan Liu; ahlwk2000-2004 @163.com;

[†]Ling Zhang, Donglan Liu contributed equally to this work.

For peer review only - http://bmjopen.bmj.com/site/about/guidelines.xhtml

Abstract

Objectives: Human metapneumovirus (HMPV) is one of the most important respiratory viral pathogens among infants and children worldwide. To analyze epidemiologic and clinical characteristics of HMPV, patients hospitalized with acute respiratory illness (ARI) were studied in Guangzhou, southern China.

Study design: A cross-sectional study.

Setting: Two hospitals in Guangzhou.

Participants and methods: Throat swabs (n=5133) were collected and tested from pediatric patients (≤14 years) hospitalized with ARI over a three-year period, and 101 of 103 (98.1) HMPV-positive patients' clinical presentations were recorded for further analysis.

Results: Of the 5,133 patients, 103 (2.0%) were positive for HMPV. More frequency of HMPV infection occurred in patients ≤5 year-old (2.2%, 98/4399) than in patients >5 year-old (0.7%, 5/734) (*p*=0.004), and mostly distributed in children with the age groups of 3–6 months (2.4%, 10/423), >6–12 months (2.2%, 30/1342), >1–2 years (2.8%, 21/752) and >2–5 years (2.3%, 32/1403) (*p*=0.865). Two seasonal HMPV frequency peaks were found every year, and mainly occurred in spring and early summer. Nineteen of 103 (18.4%) HMPV-positive patients had co-infection with other pathogens, and most common co-pathogen was respiratory syncytial virus (36.8%, 7/19). HMPV infection led to a wide spectrum of symptoms, and the main symptoms included cough (100.0%, 101/101), abnormal pulmonary breath sound (91.1%, 92/101), fever (88.1%, 89/101), expectoration (77.2%, 78/101), bronchopn-

For peer review only - http://bmjopen.bmj.com/site/about/guidelines.xhtml

eumonia (55.4%, 56/101), coryza (50.5%, 51/101) and wheezing (46.5%, 47/101). More fever (≥38˚C) cases (71.6%, 76/83) were found among mono-infected patients than co-infected patients (72.2%, 13/18) (p=0.037). While diarrhea was found more frequently in HMPV co-infected patients (22.2%, 4/18) than mono-infected patients (3.6%, 3/83) (p=0.018).

Conclusions: This study provides valuable insight into HMPV epidemiology and clinical characteristics, which may be helpful in the control and prevention of infectious diseases.

Strengths and limitations of this study

1 .The study investigate the epidemiological and clinical features of human metapneumovirus (HMPV) infection in pediatric patients hospitalized with acute respiratory illness, which may aid in diagnosis, control and prevention of infectious diseases.

2. The study of relatively large sample size is helpful to the accurate study of HMPV characteristics.

3. The study had some limitations: the characteristics of HMPV-negative patients were not further analyzed, and bacterial pathogens were not detected in HMPV-infection patients. These defects may affect our comprehensive understanding of HMPV infection in epidemiological and clinical features.

For peer review only - http://bmjopen.bmj.com/site/about/guidelines.xhtml

Introduction

Human metapneumovirus (HMPV) is a nonsegmented, negative-sense single-stranded RNA virus, which belongs to the *Paramyxoviridae* family, *Pneumovirinae* subfamily, and *Metapneumovirus* genus.[1] HMPV was first discovered in 2001 in the Netherlands and was isolated from a pediatric patient with acute respiratory illness (ARI).[2] Since then, HMPV has been associated with acute respiratory disease in individuals of all ages worldwide, but it is more common in children, elderly, and immunocompromised adults.[3-5] Young children aged ≤5 years seem to be particularly susceptible to HMPV infection. In previous studies,[6-12] which mostly detected in developed countries, approximately 2.5–11.3% of respiratory samples were positive for HMPV in children ≤5 years and 90% of individuals have positive serology for HMPV by 5 years of age.[13]

HMPV causes a variety of clinical symptoms ranging from a mild upper respiratory tract infection (URTI) to life-threatening lower respiratory tract infection (LRTI).[3 14-16] And until now, there is no effective vaccine and drug treatment for HMPV. Thus, more studies are needed all over the world, especially in developing countries, where only a limited number of reports about HMPV.

In this study, we investigated the epidemiological and clinical features of HMPV infection in pediatric patients (≤14 years) hospitalized with ARI in Guangzhou, southern China, between July 2013 and June 2016 using real-time PCR. The findings of this study will be helpful in understanding the distribution of HMPV in subtropical region and providing the data for developing effective control and prevention

For peer review only - http://bmjopen.bmj.com/site/about/guidelines.xhtml

83 strategies. Our results also provide valuable insight into the clinical features of HMPV,

84 which will be helpful in early clinical diagnosis.

For peer review only

For peer review only - http://bmjopen.bmj.com/site/about/guidelines.xhtml

Methods

Study design and respiratory sample collection

This was a cross-sectional study at The First Affiliated Hospital of Guangzhou Medical University and Sun Yat-Sen Memorial Hospital in Guangzhou. Samples in this study were taken as part of standard care. Throat swab samples (n=5133) from pediatric patients (≤14 years) hospitalized with ARI (presenting with at least two of the following symptoms: cough, pharyngeal discomfort, nasal obstruction, coryza, sneeze, dyspnoea or diagnosed with pneumonia by chest radiography during the previous week) were collected at two hospitals between July 2013 and June 2016. The samples were collected with established clinical protocols.[17] The samples were refrigerated at 2–8°C in viral transport medium, transported on ice to the State Key Laboratory of Respiratory Diseases, and analyzed immediately or stored at -80°C before analysis as previously described.[18]

The patients' clinical symptoms were extracted from their medical records using designed presentation cards and were retrospectively categorized into the following four groups: URTI, LRTI, systemic influenza-like symptoms, and gastrointestinal illness. Patients with nasal obstruction, coryza, sneezing, coughing, pharyngeal discomfort, or hoarseness were categorized as having URTI. Patients with pneumonia, bronchiolitis, increased lung markings, dyspnea, or an abnormal pulmonary breath sound were categorized as having LRTI. Patients with high fever (≥38°C), chills, dizziness, headache, myalgia, or debility were categorized as having systemic influenza-like symptoms. Patients with vomiting, poor appetite, or diarrhea

For peer review only - http://bmjopen.bmj.com/site/about/guidelines.xhtml

were categorized as having gastrointestinal illness. Some patients were assigned to several clinical presentation groups. Patients with incomplete clinical data were excluded from this analysis. Increased lung markings, bronchopneumonia, pneumonia, and bronchiolitis were diagnosed by chest radiography. Abnormal pulmonary breath sounds included phlegmatic rales, wheezy rales, bubbling rales, and moist rales. Other clinical symptoms were identified by a general medical examination and clinical descriptions as previously reported.[19]

Real-time RT-PCR for HMPV detection

RNA were extracted from the respiratory samples with the QIAamp Viral RNA Mini Kit (Qiagen, Shanghai, China), respectively, according to the manufacturer's protocols. HMPV was determined by using TaqMan Real-time PCR assays, as previously reported,[18] using kits from Guangzhou HuYanSuo Medical Technology Co., Ltd according to the manufacturer's protocols.

Detection of common respiratory pathogens in HMPV-positive patients

HMPV-positive samples were simultaneously tested by using TaqMan Real-time PCR assays for the following 17 respiratory pathogens : respiratory syncytial virus (RSV), parainfluenza virus types 1–4 (PIV1–4), influenza A and B viruses (InfA, InfB), adenovirus (ADV), enterovirus (EV), human coronaviruses (HCoV-229E, HCoV-OC43, HCoV-NL63, HCoV-HKU1), human rhinovirus (HRV), human bocavirus (HBoV), *Mycoplasma pneumoniae* (MP), and *Chlamydophila pneumoniae* (CP). The testing

For peer review only - http://bmjopen.bmj.com/site/about/guidelines.xhtml

procedure was conducted using kits from Guangzhou HuYanSuo Medical Technology Co., Ltd as previously described.[18]

Statistical analysis

All statistical analyses were performed with SPSS statistical software (version 19.0; SPSS Inc., Chicago, IL, USA). To compare categorical data, the χ^2 test and Fisher's exact test were used as appropriate. All tests were two-tailed, and $p<0.05$ was considered statistically significant.

For peer review only - http://bmjopen.bmj.com/site/about/guidelines.xhtml

Results

Detection of HMPV from patients with ARI

In total, 5133 pediatric patients (≤14 years) hospitalized with ARI between July 2013 and June 2016 were enrolled in this study. The male-to-female ratio was 1.75 (3269:1864) and the median age was 2.76 years (interquartile range, 0.75–4.00 years). Of the 5133 patients, 103 (2.0%) were positive for HMPV. The male-to-female ratio was 1.86:1 (67:36) in HMPV-positive patients and 1.75:1 (3202:1828) in HMPV-negative patients (*p*=0.771). The median age of HMPV-positive patients was 1.95 years (interquartile range, 0.83–3.00 years).

Co-infection with common respiratory pathogens

In this study, we tested HMPV-positive patients for 17 other common respiratory pathogens. Of the 103 HMPV-positive patients, 84 (81.6%) patients had HMPV mono-infection and 19 (18.4%) patients had HMPV co-infection with ≥1 other pathogen. The male-to-female ratio was 2:1 (56:28) in HMPV mono-infected patients and 1.38:1 (11:8) in HMPV co-infected patients (*p*=0.469). Nine of these 17 pathogens (52.9%) were detected, and the most frequent co-pathogens were RSV (36.8%, 7/19), HCoV-OC43 (15.8%, 3/19), MP (15.8%, 3/19), and HBoV (15.8%, 3/19) (**Table 1**).

Age distributions of HMPV-positive patients

Overall, a significant difference were found between the distribution of HMPV-positive patients aged >5–14 years (0.7%, 5/734) and those aged ≤5 years

For peer review only - http://bmjopen.bmj.com/site/about/guidelines.xhtml

(2.2%, 98/4399) (p=0.004), for detail, the patients were divided into six age groups: 0–3 months, >3–6 months, >6–12 months, >1–2 years, >2–5 years, and >5–14 years. The distribution of HMPV-infected patients in each group was significantly different (p=0.03) (**Figure 1**). HMPV infection most frequently occurred in children aged >1–2 years (2.8%, 21/752) (**Figure 1**). However, there were no significant difference in frequency of HMPV infection among patients aged >1–2 years, >3–6 months (2.4%, 10/423), >6–12 months (2.2%, 30/1342), and >2–5 years (2.3%, 32/1403) (p=0.865).

Seasonal distribution of HMPV infection

In general, the frequency of HMPV infections peaked twice every year (**Figure 2**). Large peaks in the HMPV detection rate occurred in March 2014 (8%, 16/200), May 2015 (7.6%, 8/105), and February 2016 (8.7%, 9/103). Small peaks in the HMPV detection rates occurred in November 2014 (1.7%, 2/121), September 2015 (2.8%, 2/72), and May 2016 (1.5%, 2/135) (**Figure 2**).

Clinical presentations of HMPV-positive patients

We analyzed the clinical presentations of 101 of 103 (98.1%) HMPV-positive patients. Patients with incomplete clinical data were excluded from this analysis (n=2). The main symptoms of HMPV infection included cough (100.0%, 101/101), abnormal pulmonary breath sound (91.1%, 92/101), fever (88.1%, 89/101), expectoration (77.2%, 78/101), bronchopneumonia (55.4%, 56/101), coryza (50.5%, 51/101) and wheezing (46.5%, 47/101) (**Table 2**). We also compared the clinical features of

For peer review only

patients with HMPV mono-infection and co-infections, and significant differences were found in patients with fever (≥38˚C) (*p*=0.037) and diarrhea (*p*=0.018), which observed in 91.6% (76/83), 3.6% (3/83) patients with single infection and 72.2% (13/18), 22.2% (4/18) patients with co-infection, respectively (**Table 2**).

For peer review only

Discussion

Viruses are the most frequent cause of respiratory infection.[20] HMPV has been recognized as an important respiratory pathogen causing ARI since its discovery in 2001. It has been reported that HMPV was responsible for approximately 5–10% of hospitalizations of children suffering from ARI,[14] which has led to considerable clinical and economic burden worldwide. HMPV detection rates vary according to geographic location, and the incidence of HMPV may show seasonal or annual patterns in the same area. In previous studies,[21-30] the HMPV detection rate was approximately 1–17% of ARI. In this 3-year study, 103 of 5133 (2.0%) patients were positive for HMPV, which is similar to previous reports of HMPV in patients with ARI in southern China[31] and Japan.[32]

HMPV is commonly found in the pediatric population, with children under 5 year-old most susceptible to and children under 2 year-old at the greatest risk for serious HMPV infections.[15] In our study, more frequency of HMPV infection occurred in patients ≤5 year-old (2.2%, 98/4399) than in patients >5 year-old (0.7%, 5/734) (p=0.004), which is consistent with most previous studies.[3 30 33 34] In detail, HMPV infection were found high frequency in children with the age groups of 3–6 months, >6–12 months, >1–2 years and >2–5 years (p=0.865) (**Figure 1**). Therefore, children of this age need more attention to prevent HMPV infection. Moreover, our findings suggested a male predominance, there was no significant difference in the HMPV positivity rate between males and females (p=0.771), which is consistent with previous studies.[35 36]

For peer review only - http://bmjopen.bmj.com/site/about/guidelines.xhtml

HMPV has a typical seasonal distribution. The activity of HMPV is largely affected by different climate factors, which are locally present. The activity of HMPV in temperate climates peaks at the end of winter or in early spring.[37 38] In this study, seasonal peaks of HMPV infection were detected in February 2016, March 2014, and May 2015 (**Figure 2**). This finding indicates that in Guangzhou, HMPV circulates primarily during the spring and early summer, as previously reported in other subtropical areas.[22 39] Furthermore, we also found small peaks in November 2014, September 2015, and May 2016 (**Figure 2**). Generally, the seasonal distribution pattern of HMPV overlaps that of RSV in this area.[19] These data might be useful for control and prevention strategies of HMPV.

HMPV co-infection with other respiratory pathogens has been reported in many studies, including RSV,[40] InfA,[41] InfB,[42] PIV,[43] ADV,[43 44] HBoV,[45] HCoV ,[43 46] RV,[43] EV,[43] MP,[41] and CP.[41] In this study, most HMPV-positive patients had mono-infection (81.6%, 84/103), and there was no significant difference in the HMPV positivity rate according to sex between HMPV mono- and co-infected patients (p=0.469). The co-infection rate was 18.4% (19/103), and the most frequently detected co-pathogen was RSV (36.8%, 7/19) (**Table 1**), which might be due to the overlapping seasonal distribution of HMPV and RSV.[47] Co-infection with HCoV-OC43, MP, HBoV, ADV, RV, InfA, PIV2, and PIV3 occurred at rates >5%, suggesting a broad range of co-pathogens in HMPV infection (**Table 1**).

HMPV most commonly causes URTI and LRTI in young children. The clinical manifestations of HMPV infection are similar to those of RSV infection, especially in

For peer review only - http://bmjopen.bmj.com/site/about/guidelines.xhtml

young children,[48-50] and HMPV has been reported that the most frequent diagnoses of children hospitalized with HMPV infection were pneumonitis and bronchiolitis.[3 51 52] In some studies, bronchiolitis and recurrent wheezing/pneumonia were the main clinical diagnosis,[7 22 53 54] while in other studies, bronchopneumonia, bronchiolitis, and bronchial asthma exacerbation were the main clinical diagnosis.[34] In this study, we analyzed the clinical presentations of 101 HMPV-positive patients (**Table 2**), and 55.4%, 7.9%, and 10.9% of patients were diagnosed with bronchopneumonia, bronchiolitis, and pneumonia by chest radiography, respectively. Of all clinical presentations, cough (100.0%), abnormal pulmonary breath sound (91.1%), fever (88.1%), expectoration (77.2%), bronchopneumonia (55.4%), coryza (50.5%), and wheezing (46.5%) occurred frequently in HMPV-positive patients (Table 2), similar to those reported previously.[16 29 52 55]

When we compared the clinical features between HMPV mono- and co-infected patients, more fever cases (p=0.037) were found among mono-infected patients than co-infected patients, suggesting that fever was the key clinical presentation caused by HMPV infection (**Table 2**). Diarrhea was found more frequently in HMPV co-infected patients than mono-infected patients (p=0.018), suggested that diarrhea is not the main manifestation of HMPV infection, but may be caused by other pathogens. No significant difference was found in the other clinical presentations between HMPV mono- and co-infected patients, similar to the results of a previous study.[51] In this study, most frequent co-pathogen in HMPV-positive patients were RSV (36.8%) (**Table 1**). Whether HMPV/RSV co-infection is more severe than

For peer review only - http://bmjopen.bmj.com/site/about/guidelines.xhtml

the respective mono-infections remains unclear. A previous study showed that children with HMPV/RSV co-infection were more likely to develop pneumonia, however, disease severity was not increased.[53] Conversely, Semple et al.[56] reported that HMPV/RSV co-infection can cause more severe bronchiolitis in patients. In our study, no significant difference in the pneumonia rate was observed between HMPV mono- and co-infected patients. It has been confirmed that a history of prematurity, particular age groups, and the presence of chronic diseases increases the risk of severe LRTI among HMPV- and RSV-infected children.[51] The clinical manifestations of HMPV infection are complex and diverse; the data in this work might be helpful in the diagnosis of HMPV. In this study, the characteristics of HMPV-negative patients were not further analyzed, and bacterial pathogens were not detected in HMPV-infection patients. These defects may affect our comprehensive understanding of HMPV infection in epidemiological and clinical features.

For peer review only - http://bmjopen.bmj.com/site/about/guidelines.xhtml

264 **Conclusions**

265 In this study, we analyzed the epidemiology and clinical manifestations of HMPV infection in hospitalized children under 14 years old over a three-year period in Guangzhou, China. Our results provide valuable insight into the epidemiology and clinical features of HMPV infection in subtropical region, which may be helpful in diagnosis, prevention of HMPV infection.

For peer review only

Acknowledgements

We thank the study volunteers for their generous participation. We thank Yinghua Zhou, Haiping Huang, Jing Zhang, Jing Ma for technical assistance. We thank Professor Xiaofeng Li and Dr. Zhengshi Lin for paper polishing.

Contributors

RZ, WKL, LZ and DLL designed the study. LZ, DLL, WKL, SYQ, DX, XL and LTT performed pathogen testing. DHC and WPT collected clinical data. All authors participated in the data analysis. LZ, DLL, RZ and WKL drafted the manuscript. All authors read and approved the final version of this manuscript.

Funding

This study was supported by The State Major Infectious Disease Research Program (RZ) (2017ZX10103011-003), National Natural Science Foundation of China (WKL) (31500143), Guangzhou Science and Technology Program key projects (RZ) (201508020252). The funders had no role in study design, data collection and analysis, decision to publish, or preparation of the manuscript.

Competing interests

None declared.

Ethics approval

For peer review only - http://bmjopen.bmj.com/site/about/guidelines.xhtml

The study was approved by The First Affiliated Hospital of Guangzhou Medical University Ethics Committee. Informed written consent was obtained from parents or legal guardians.

Data sharing statement

No additional data are available.

For peer review only

For peer review only - http://bmjopen.bmj.com/site/about/guidelines.xhtml

REFERENCES

1. van den Hoogen BG, Herfst S, Sprong L, et al. Antigenic and genetic variability of human metapneumoviruses. *Emerg Infect Dis* 2004;10(4):658-66. doi: 10.3201/eid1004.030393
2. van den Hoogen BG, de Jong JC, Groen J, et al. A newly discovered human pneumovirus isolated from young children with respiratory tract disease. *Nat Med* 2001;7(6):719-24. doi: 10.1038/89098
3. Boivin G, Abed Y, Pelletier G, et al. Virological features and clinical manifestations associated with human metapneumovirus: a new paramyxovirus responsible for acute respiratory-tract infections in all age groups. *J Infect Dis* 2002;186(9):1330-4. doi: 10.1086/344319
4. Falsey AR, Walsh EE. Viral pneumonia in older adults. *Clin Infect Dis* 2006;42(4):518-24. doi: 10.1086/499955
5. Kahn JS. Epidemiology of human metapneumovirus. *Clin Microbiol Rev* 2006;19(3):546-57. doi: 10.1128/CMR.00014-06
6. Davis CR, Stockmann C, Pavia AT, et al. Incidence, Morbidity, and Costs of Human Metapneumovirus Infection in Hospitalized Children. *J Pediatric Infect Dis Soc* 2016;5(3):303-11. doi: 10.1093/jpids/piv027
7. Edwards KM, Zhu Y, Griffin MR, et al. Burden of human metapneumovirus infection in young children. *N Engl J Med* 2013;368(7):633-43. doi: 10.1056/NEJMoa1204630
8. Foulongne V, Guyon G, Rodiere M, et al. Human metapneumovirus infection in young children hospitalized with respiratory tract disease. *Pediatr Infect Dis J* 2006;25(4):354-9. doi: 10.1097/01.inf.0000207480.55201.f6
9. Heikkinen T, Osterback R, Peltola V, et al. Human metapneumovirus infections in children. *Emerg Infect Dis* 2008;14(1):101-6. doi: 10.3201/eid1401.070251
10. Nicholson KG, McNally T, Silverman M, et al. Rates of hospitalisation for influenza, respiratory syncytial virus and human metapneumovirus among infants and young children. *Vaccine* 2006;24(1):102-8. doi: 10.1016/j.vaccine.2005.02.004
11. Sloots TP, Mackay IM, Bialasiewicz S, et al. Human metapneumovirus, Australia, 2001-2004. *Emerg Infect Dis* 2006;12(8):1263-6. doi: 10.3201/eid1208.051239
12. Williams JV, Edwards KM, Weinberg GA, et al. Population-based incidence of human metapneumovirus infection among hospitalized children. *J Infect Dis* 2010;201(12):1890-8. doi: 10.1086/652782
13. Ebihara T, Endo R, Kikuta H, et al. Seroprevalence of human metapneumovirus in Japan. *J Med Virol* 2003;70(2):281-3. doi: 10.1002/jmv.10391
14. Panda S, Mohakud NK, Pena L, et al. Human metapneumovirus: review of an important respiratory pathogen. *Int J Infect Dis* 2014;25:45-52. doi: 10.1016/j.ijid.2014.03.1394
15. Broor S, Bharaj P, Chahar HS. Human metapneumovirus: a new respiratory pathogen. *J Biosci* 2008;33(4):483-93.
16. Jain B, Singh AK, Dangi T, et al. High prevalence of human metapneumovirus subtype B in cases presenting as severe acute respiratory illness: an experience at tertiary care hospital. *Clin Respir J* 2014;8(2):225-33. doi: 10.1111/crj.12064
17. Liu WK, Liu Q, Chen DH, et al. Epidemiology and clinical presentation of the four human parainfluenza virus types. *BMC Infect Dis* 2013;13:28. doi: 10.1186/1471-2334-13-28
18. Liu WK, Chen DH, Liu Q, et al. Detection of human bocavirus from children and adults with acute respiratory tract illness in Guangzhou, southern China. *BMC Infect Dis* 2011;11:345. doi:

For peer review only - http://bmjopen.bmj.com/site/about/guidelines.xhtml

10.1186/1471-2334-11-345

19. Liu W, Chen D, Tan W, et al. Epidemiology and Clinical Presentations of Respiratory Syncytial Virus Subgroups A and B Detected with Multiplex Real-Time PCR. *PLoS One* 2016;11(10):e0165108.DOI: 10.1371/journal.pone.08. doi: 10.1371/journal.pone.0165108
20. Williams BG, Gouws E, Boschi-Pinto C, et al. Estimates of world-wide distribution of child deaths from acute respiratory infections. *Lancet Infect Dis* 2002;2(1):25-32.
21. Escobar C, Luchsinger V, de Oliveira DB, et al. Genetic variability of human metapneumovirus isolated from Chilean children, 2003-2004. *J Med Virol* 2009;81(2):340-4. doi: 10.1002/jmv.21399
22. Garcia-Garcia ML, Calvo C, Rey C, et al. Human metapnuemovirus infections in hospitalized children and comparison with other respiratory viruses. 2005-2014 prospective study. *PLoS One* 2017;12(3):e0173504.DOI: 10.1371/journal.pone.04. doi: 10.1371/journal.pone.0173504
23. Ljubin-Sternak S, Santak M, Cepin-Bogovic J, et al. Detection of genetic lineages of human metapneumovirus in Croatia during the winter season 2005/2006. *J Med Virol* 2008;80(7):1282-7. doi: 10.1002/jmv.21196
24. McCracken JP, Arvelo W, Ortiz J, et al. Comparative epidemiology of human metapneumovirus- and respiratory syncytial virus-associated hospitalizations in Guatemala. *Influenza Other Respir Viruses* 2014;8(4):414-21. doi: 10.1111/irv.12251
25. Nor'e SS, Sam IC, Mohamad Fakri EF, et al. Phylogenetic analysis of human metapneumovirus among children with acute respiratory infections in Kuala Lumpur, Malaysia. *Trop Biomed* 2014;31(3):562-6.
26. Ordas J, Boga JA, Alvarez-Arguelles M, et al. Role of metapneumovirus in viral respiratory infections in young children. *J Clin Microbiol* 2006;44(8):2739-42. doi: 10.1128/JCM.00164-06
27. Rafiefard F, Yun Z, Orvell C. Epidemiologic characteristics and seasonal distribution of human metapneumovirus infections in five epidemic seasons in Stockholm, Sweden, 2002-2006. *J Med Virol* 2008;80(9):1631-8. doi: 10.1002/jmv.21242
28. Rodriguez PE, Adamo MP, Paglini MG, et al. [Monoinfection of human Metapneumovirus in Cordoba: first clinical and epidemiological research in children with respiratory infection in 2011]. *Rev Fac Cien Med Univ Nac Cordoba* 2016;73(3):170-75.
29. Zeng SZ, Xiao NG, Zhong LL, et al. Clinical features of human metapneumovirus genotypes in children with acute lower respiratory tract infection in Changsha, China. *J Med Virol* 2015;87(11):1839-45. doi: 10.1002/jmv.24249
30. Zhu RN, Qian Y, Zhao LQ, et al. Characterization of human metapneumovirus from pediatric patients with acute respiratory infections in a 4-year period in Beijing, China. *Chin Med J (Engl)* 2011;124(11):1623-8.
31. Cai XY, Wang Q, Lin GY, et al. Respiratory virus infections among children in South China. *J Med Virol* 2014;86(7):1249-55. doi: 10.1002/jmv.23931
32. Matsuzaki Y, Itagaki T, Abiko C, et al. Clinical impact of human metapneumovirus genotypes and genotype-specific seroprevalence in Yamagata, Japan. *J Med Virol* 2008;80(6):1084-9. doi: 10.1002/jmv.21194
33. Barbosa Ramirez J, Pulido Dominguez P, Rey Benito G, et al. Human respiratory syncytial virus and metapneumovirus in patients with acute respiratory infection in Colombia, 2000 - 2011. *Rev Panam Salud Publica* 2014;36(2):101-9.

For peer review only

34. Zhang C, Du LN, Zhang ZY, et al. Detection and genetic diversity of human metapneumovirus in hospitalized children with acute respiratory infections in Southwest China. *J Clin Microbiol* 2012;50(8):2714-9. doi: 10.1128/JCM.00809-12
35. McAdam AJ, Hasenbein ME, Feldman HA, et al. Human metapneumovirus in children tested at a tertiary-care hospital. *J Infect Dis* 2004;190(1):20-6. doi: 10.1086/421120
36. Boivin G, De Serres G, Cote S, et al. Human metapneumovirus infections in hospitalized children. *Emerg Infect Dis* 2003;9(6):634-40. doi: 10.3201/eid0906.030017
37. Maggi F, Pifferi M, Vatteroni M, et al. Human metapneumovirus associated with respiratory tract infections in a 3-year study of nasal swabs from infants in Italy. *J Clin Microbiol* 2003;41(7):2987-91.
38. van den Hoogen BG, van Doornum GJ, Fockens JC, et al. Prevalence and clinical symptoms of human metapneumovirus infection in hospitalized patients. *J Infect Dis* 2003;188(10):1571-7. doi: 10.1086/379200
39. Peiris JS, Tang WH, Chan KH, et al. Children with respiratory disease associated with metapneumovirus in Hong Kong. *Emerg Infect Dis* 2003;9(6):628-33. doi: 10.3201/eid0906.030009
40. Greensill J, McNamara PS, Dove W, et al. Human metapneumovirus in severe respiratory syncytial virus bronchiolitis. *Emerg Infect Dis* 2003;9(3):372-5. doi: 10.3201/eid0903.020289
41. Lin PY, Lin TY, Huang YC, et al. Human metapneumovirus and community-acquired pneumonia in children. *Chang Gung Med J* 2005;28(10):683-8.
42. Ghattas C, Mossad SB. Fatal human metapneumovirus and influenza B virus coinfection in an allogeneic hematopoietic stem cell transplant recipient. *Transpl Infect Dis* 2012;14(5):E41-3.DOI: 10.1111/j.399-3062.2012.00766.x. doi: 10.1111/j.1399-3062.2012.00766.x
43. Fathima S, Lee BE, May-Hadford J, et al. Use of an innovative web-based laboratory surveillance platform to analyze mixed infections between human metapneumovirus (hMPV) and other respiratory viruses circulating in Alberta (AB), Canada (2009-2012). *Viruses* 2012;4(11):2754-65. doi: 10.3390/v4112754
44. Kaida A, Kubo H, Goto K, et al. Co-infection of human metapneumovirus with adenovirus or respiratory syncytial virus among children in Japan. *Microbiol Immunol* 2007;51(7):679-83.
45. Pilger DA, Cantarelli VV, Amantea SL, et al. Detection of human bocavirus and human metapneumovirus by real-time PCR from patients with respiratory symptoms in Southern Brazil. *Mem Inst Oswaldo Cruz* 2011;106(1):56-60.
46. Esposito S, Daleno C, Prunotto G, et al. Impact of viral infections in children with community-acquired pneumonia: results of a study of 17 respiratory viruses. *Influenza Other Respir Viruses* 2013;7(1):18-26. doi: 10.1111/j.1750-2659.2012.00340.x
47. Kahn JS. Newly discovered respiratory viruses: significance and implications. *Curr Opin Pharmacol* 2007;7(5):478-83. doi: 10.1016/j.coph.2007.07.004
48. Wolf DG, Greenberg D, Kalkstein D, et al. Comparison of human metapneumovirus, respiratory syncytial virus and influenza A virus lower respiratory tract infections in hospitalized young children. *Pediatr Infect Dis J* 2006;25(4):320-4. doi: 10.1097/01.inf.0000207395.80657.cf
49. Wilkesmann A, Schildgen O, Eis-Hubinger AM, et al. Human metapneumovirus infections cause similar symptoms and clinical severity as respiratory syncytial virus infections. *Eur J*

For peer review only - http://bmjopen.bmj.com/site/about/guidelines.xhtml

Pediatr 2006;165(7):467-75. doi: 10.1007/s00431-006-0105-4

50. Adams O, Weis J, Jasinska K, et al. Comparison of human metapneumovirus, respiratory syncytial virus and Rhinovirus respiratory tract infections in young children admitted to hospital. *J Med Virol* 2015;87(2):275-80. doi: 10.1002/jmv.24025

51. Moe N, Krokstad S, Stenseng IH, et al. Comparing Human Metapneumovirus and Respiratory Syncytial Virus: Viral Co-Detections, Genotypes and Risk Factors for Severe Disease. *PLoS One* 2017;12(1):e0170200.DOI: 10.1371/journal.pone.00. doi: 10.1371/journal.pone.0170200

52. Yan XL, Li YN, Tang YJ, et al. Clinical characteristics and viral load of respiratory syncytial virus and human metapneumovirus in children hospitaled for acute lower respiratory tract infection. *J Med Virol* 2017;89(4):589-97. doi: 10.1002/jmv.24687

53. Caracciolo S, Minini C, Colombrita D, et al. Human metapneumovirus infection in young children hospitalized with acute respiratory tract disease: virologic and clinical features. *Pediatr Infect Dis J* 2008;27(5):406-12. doi: 10.1097/INF.0b013e318162a164

54. Williams JV, Harris PA, Tollefson SJ, et al. Human metapneumovirus and lower respiratory tract disease in otherwise healthy infants and children. *N Engl J Med* 2004;350(5):443-50. doi: 10.1056/NEJMoa025472

55. Schuster JE, Khuri-Bulos N, Faouri S, et al. Human Metapneumovirus Infection in Jordanian Children: Epidemiology and Risk Factors for Severe Disease. *Pediatr Infect Dis J* 2015;34(12):1335-41. doi: 10.1097/INF.0000000000000892

56. Semple MG, Cowell A, Dove W, et al. Dual infection of infants by human metapneumovirus and human respiratory syncytial virus is strongly associated with severe bronchiolitis. *J Infect Dis* 2005;191(3):382-6. doi: 10.1086/426457

For peer review only

For peer review only - http://bmjopen.bmj.com/site/about/guidelines.xhtml

Table 1. Distribution of co-pathogens in 19 HMPV-positive patients

Co-pathogens[a]	Patient No. (%)
RSV/HMPV	7(36.8)
HCoV-OC43/ HMPV	3(15.8)
MP/ HMPV	3(15.8)
HBoV/ HMPV	3(15.8)
ADV/ HMPV	2(10.5)
HRV/ HMPV	2(10.5)
InfA/ HMPV	2(10.5)
PIV2/ HMPV	1(5.3)
PIV3/ HMPV	1(5.3)

[a]HMPV-positive patients were tested for 17 common respiratory pathogens.

InfB, PIV1, PIV4, EV, HCoV-HKU1, HCoV-229E, HCoV-NL63, CP were not detected.

Percentages sum to >100% because some patients were co-infected with more than two viruses.

For peer review only

Table 2. Clinical presentations in HMPV-positive patients

Charactristics	Total HMPV infection (n=101)	Patients with single HMPV and co-pathogens		
		Single HMPV (n = 83)	Co-pathogens (n= 18)	p value[b]
**URTI**				
Nasal obstruction	40(39.6)	34(41.0)	6(33.3)	0.605
Coryza	51(50.5)	44(53.0)	7(38.9)	0.309
Sneeze	1(1.0)	1(1.2)	0(0)	–[c]
Cough	101(100)	83(100)	18(100)	–[d]
Expectoration	78(77.2)	66(79.5)	12(66.7)	0.351
Pharyngeal discomfort	2(2.0)	2(2.4)	0(0)	–[c]
Hoarseness	1(1.0)	1(1.2)	0(0)	–[c]
**LRTI**				
Abnormal pulmonary breath sound [a]	92(91.1)	75(90.4)	17(94.4)	0.582
Increasing lung markings	6(6.0)	4(4.8)	2(11.1)	0.29
Bronchiolitis	8(7.9)	7(8.4)	1(5.6)	0.682
Pneumonia	11(10.9)	10(12.1)	1(5.6)	0.684
bronchopneumonia	56(55.4)	46(55.4)	10(55.6)	0.992
wheezing	47(46.5)	40(48.2)	7(38.9)	0.473
anhelation	24(23.8)	21(25.3)	3(16.7)	0.551
**Systemic influenza-like symptoms**				
Fever (≥38˚C)	89(88.1)	76(91.6)	13(72.2)	**0.037**
Chill	7(6.9)	6(7.2)	1(5.6)	0.8
Debilitation	11(10.9)	11(13.3)	0(0)	–[c]
**Gastrointestinal illness**				
Vomiting	21(20.8)	16(19.3)	5(27.8)	0.522
Poor appetite	19(18.8)	17(20.5)	2(11.1)	0.513
Diarrhea	7(7.0)	3(3.6)	4(22.2)	**0.018**

Data are presented as No. (%) of each group.

Percentages sum to >100% because some patients had >1 clinical presentations.

[a]Including phlegmatic rales, wheezing rales, bubbling rales, and moist rales.

[b]Two-tailed χ^2 test comparing the distribution of each illness or diagnosis between HMPV mono- and co-infected patients.

[c]Not tested because the number of positive samples obtained was small.

[d]Not tested because the rate of positive samples obtained was 100%.

For peer review only

For peer review only - http://bmjopen.bmj.com/site/about/guidelines.xhtml

Figure 1

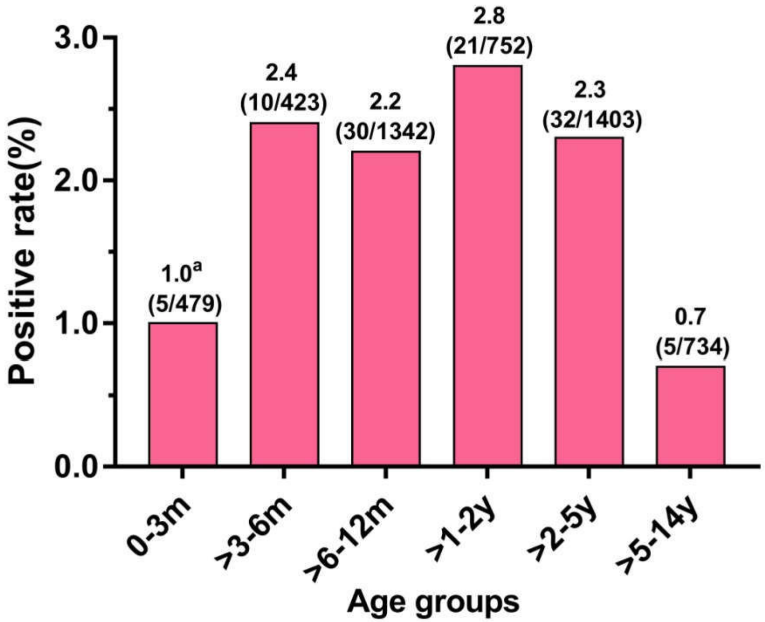

Figure 1 Age distributions of patients with HMPV.
[a]Date were presented as positive rate(positive number/total number) in each group; m: month(s); y: year(s).

Figure 2

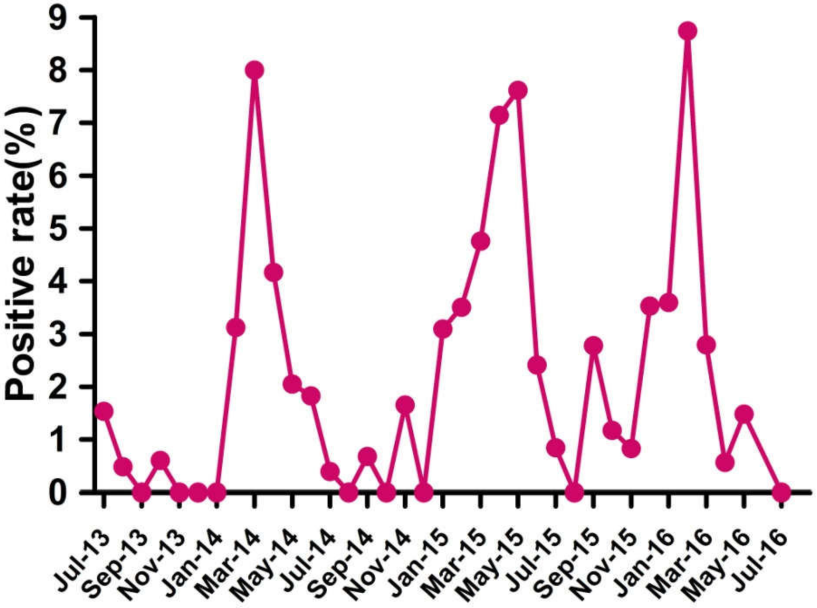

Figure 2 Seasonal distribution of HMPV infection in pediatric patients hospitalized with acute respiratory infection from July 2013 to June 2016 in Guangzhou.

STROBE 2007 (v4)Statement—Checklist of items that should be included in reports of *cross-sectional studies*

Section/Topic	Item #	Recommendation	Reported on page #
**Title and abstract**	1	(*a*) Indicate the study's design with a commonly used term in the title or the abstract	1
		(*b*) Provide in the abstract an informative and balanced summary of what was done and what was found	2-3
**Introduction**			
Background/rationale	2	Explain the scientific background and rationale for the investigation being reported	4
Objectives	3	State specific objectives, including any prespecified hypotheses	4-5
**Methods**			
Study design	4	Present key elements of study design early in the paper	6
Setting	5	Describe the setting, locations, and relevant dates, including periods of recruitment, exposure, follow-up, and data collection	6
Participants	6	(*a*) Give the eligibility criteria, and the sources and methods of selection of participants	6
Variables	7	Clearly define all outcomes, exposures, predictors, potential confounders, and effect modifiers. Give diagnostic criteria, if applicable	6-7
Data sources/ measurement	8*	For each variable of interest, give sources of data and details of methods of assessment (measurement). Describe comparability of assessment methods if there is more than one group	7
Bias	9	Describe any efforts to address potential sources of bias	*6*
Study size	10	Explain how the study size was arrived at	6
Quantitative variables	11	Explain how quantitative variables were handled in the analyses. If applicable, describe which groupings were chosen and why	7-8
Statistical methods	12	(*a*) Describe all statistical methods, including those used to control for confounding	8
		(*b*) Describe any methods used to examine subgroups and interactions	8
		(*c*) Explain how missing data were addressed	7
		(*d*) If applicable, describe analytical methods taking account of sampling strategy	8
		(*e*) Describe any sensitivity analyses	
**Results**			

For peer review only

Participants	13*	(a) Report numbers of individuals at each stage of study—eg numbers potentially eligible, examined for eligibility, confirmed eligible, included in the study, completing follow-up, and analysed	9
		(b) Give reasons for non-participation at each stage	10
		(c) Consider use of a flow diagram	
Descriptive data	14*	(a) Give characteristics of study participants (eg demographic, clinical, social) and information on exposures and potential confounders	9-11
		(b) Indicate number of participants with missing data for each variable of interest	9
Outcome data	15*	Report numbers of outcome events or summary measures	9-11
Main results	16	(*a*) Give unadjusted estimates and, if applicable, confounder-adjusted estimates and their precision (eg, 95% confidence interval). Make clear which confounders were adjusted for and why they were included	9-11
		(*b*) Report category boundaries when continuous variables were categorized	9-10
		(*c*) If relevant, consider translating estimates of relative risk into absolute risk for a meaningful time period	
Other analyses	17	Report other analyses done—eg analyses of subgroups and interactions, and sensitivity analyses	
**Discussion**			
Key results	18	Summarise key results with reference to study objectives	12-15
Limitations	19	Discuss limitations of the study, taking into account sources of potential bias or imprecision. Discuss both direction and magnitude of any potential bias	15
Interpretation	20	Give a cautious overall interpretation of results considering objectives, limitations, multiplicity of analyses, results from similar studies, and other relevant evidence	12-15
Generalisability	21	Discuss the generalisability (external validity) of the study results	12-15
**Other information**			
Funding	22	Give the source of funding and the role of the funders for the present study and, if applicable, for the original study on which the present article is based	17

*Give information separately for cases and controls in case-control studies and, if applicable, for exposed and unexposed groups in cohort and cross-sectional studies.

Note: An Explanation and Elaboration article discusses each checklist item and gives methodological background and published examples of transparent reporting. The STROBE checklist is best used in conjunction with this article (freely available on the Web sites of PLoS Medicine at http://www.plosmedicine.org/, Annals of Internal Medicine at http://www.annals.org/, and Epidemiology at http://www.epidem.com/). Information on the STROBE Initiative is available at www.strobe-statement.org.

For peer review only

Epidemiology and clinical features of human metapneumovirus in children hospitalized with acute respiratory illness from 2013 to 2016: a cross-sectional study in Guangzhou, southern China

Journal:	*BMJ Open*
Manuscript ID	bmjopen-2017-019308.R1
Article Type:	Research
Date Submitted by the Author:	25-Oct-2017
Complete List of Authors:	Zhang, Ling; State Key Laboratory of Respiratory Diseases, The First Affiliated Hospital of Guangzhou Medical University, Guangzhou Medical University<br>Liu, Donglan; State Key Laboratory of Respiratory Diseases, The First Affiliated Hospital of Guangzhou Medical University, Guangzhou Medical University<br>Chen, Dehui; Department of Pediatrics, The First Affiliated Hospital of Guangzhou Medical University, Guangzhou Medical University<br>Tan, Weiping ; Sun Yat-Sen Memorial Hospital, Sun Yat-Sen University<br>Qiu, Shuyan; State Key Laboratory of Respiratory Diseases, The First Affiliated Hospital of Guangzhou Medical University, Guangzhou Medical University<br>Xu, Duo; State Key Laboratory of Respiratory Diseases, The First Affiliated Hospital of Guangzhou Medical University, Guangzhou Medical University<br>Li, Xiao; State Key Laboratory of Respiratory Diseases, The First Affiliated Hospital of Guangzhou Medical University, Guangzhou Medical University<br>Liu, Tiantian; State Key Laboratory of Respiratory Diseases, The First Affiliated Hospital of Guangzhou Medical University, Guangzhou Medical University<br>Liu, Wenkuan; State Key Laboratory of Respiratory Diseases, The First Affiliated Hospital of Guangzhou Medical University, Guangzhou Medical University<br>Zhou, Rong; State Key Laboratory of Respiratory Diseases, The First Affiliated Hospital of Guangzhou Medical University, Guangzhou Medical University
**Primary Subject Heading**:	Infectious diseases
Secondary Subject Heading:	Epidemiology, Paediatrics, Infectious diseases
Keywords:	Human metapneumovirus, Epidemiology < TROPICAL MEDICINE, Clinical feature, Acute respiratory illness

SCHOLARONE™
Manuscripts

For peer review only

Epidemiology and clinical features of human metapneumovirus in children hospitalized with acute respiratory illness from 2013 to 2016: a cross-sectional study in Guangzhou, southern China

Ling Zhang[1†], Donglan Liu[1†], Dehui Chen[2], Weiping Tan[3], Shuyan Qiu[1], Duo Xu[1], Xiao Li[1], Tiantian Liu[1], Wenkuan Liu[1]*, Rong Zhou[1]*

[1]State Key Laboratory of Respiratory Diseases, The First Affiliated Hospital of Guangzhou Medical University, Guangzhou Medical University, Guangzhou, People's Republic of China.

[2]Department of Pediatrics, The First Affiliated Hospital of Guangzhou Medical University, Guangzhou Medical University, Guangzhou, People's Republic of China.

[3]Sun Yat-Sen Memorial Hospital, Sun Yat-Sen University, Guangzhou, People's Republic of China.

*Correspondence to Wenkuan Liu; ahlwk2000-2004@163.com and Rong Zhou; zhourong@gird.cn

[†]Ling Zhang, Donglan Liu contributed equally to this work.

For peer review only - http://bmjopen.bmj.com/site/about/guidelines.xhtml

Abstract

Objectives: Human metapneumovirus (HMPV) is one of the most important respiratory viral pathogens among infants and children worldwide. Our study was designed to analyze epidemiological and clinical characteristics of HMPV present in patients hospitalized with acute respiratory illness (ARI) in Guangzhou, southern China.

Study design: A cross-sectional study.

Setting: Two tertiary hospitals in Guangzhou.

Participants and methods: Throat swabs were collected and tested from pediatric patients (≤14 years, n=5133) hospitalized with ARI over a three-year period, and 101 of 103 (98.1%) HMPV-positive patients' clinical presentations were recorded for further analysis.

Results: Of the 5133 patients, 103 (2.0%) were positive for HMPV. HMPV mostly distributed in children with the age groups of >3–6 months (2.4%, 10/423), >6–12 months (2.2%, 30/1342), >1–2 years (2.8%, 21/752) and >2–5 years (2.3%, 32/1403) (p=0.865). Two seasonal HMPV frequency peaks were found every year, and mainly occurred in spring and early summer. Nineteen of 103 (18.4%) HMPV-positive patients were co-detected with other pathogens, and the most common co-detected pathogen was respiratory syncytial virus (36.8%, 7/19). HMPV-positive patients presented a wide spectrum of symptoms, including cough (100.0%, 101/101), abnormal pulmonary breath sound (91.1%, 92/101), fever (88.1%, 89/101), expectoration (77.2%, 78/101), coryza (50.5%, 51/101) and wheezing (46.5%,

For peer review only - http://bmjopen.bmj.com/site/about/guidelines.xhtml

47/101). The major diagnosis of HMPV-positive patients was bronchopneumonia (55.4%, 56/101). Fever (≥38˚C) (91.6%, 76/83) was detected more often in single HMPV-positive patients than in co-pathogens-positive patients (72.2%, 13/18) (*p*=0.037), whereas diarrhea was presented more common in co-pathogens-positive patients (22.2%, 4/18) than single HMPV-positive patients (3.6%, 3/83) (*p*=0.018).

Conclusions: HMPV is a common respiratory pathogen in children with ARI in Guangzhou, particularly in patients from 3 months to 5 years old. HMPV epidemic has a seasonal variation. Bronchopneumonia is the major diagnosis in HMPV-positive patients.

Strengths and limitations of this study

- This is the study that lasted three years consecutively and had a large sample size (more than five thousands). Patients were enrolled from two large municipal tertiary hospitals (hospital beds ＞1000). In addition, patients enrolled in the study had a large age spanning from infancy to juveniles. Thus, our results possess representative characteristics of HMPV presence in children with ARI in this area.

- The study had some limitations: the characteristics of HMPV-negative patients were not further analyzed, and bacterial pathogens were not detected in HMPV-positive patients. Lack of these data may affect our comprehensively understanding the clinical characteristics caused by this pathogen.

For peer review only - http://bmjopen.bmj.com/site/about/guidelines.xhtml

Introduction

Human metapneumovirus (HMPV) is a nonsegmented, negative-sense single-stranded RNA virus, which belongs to the *Paramyxoviridae* family, *Pneumovirinae* subfamily, and *Metapneumovirus* genus.[1] HMPV was first discovered in 2001 in the Netherlands, and isolated from a pediatric patient with acute respiratory illness (ARI).[2] Since then, HMPV has been found in associated with acute respiratory disease in individuals of all ages worldwide. Children, elderly, and immunocompromised adults are more easily to contract the virus.[3-5] Children younger than 5 years of age seem to be particularly susceptible to HMPV. Previous studies,[6-12] which mainly conducting in developed countries, have revealed that approximately 2.5–11.3% of respiratory samples were positive for HMPV in children ≤5 years, and 90% of individuals have positive serology for HMPV by 5 years of age.[13]

HMPV causes a variety of clinical symptoms ranging from a mild upper respiratory tract infection (URTI) to life-threatening lower respiratory tract infection (LRTI).[3 14-16] However, there is so far no effective vaccine and medication either for prevention or treatment available for HMPV infection. Thus, it is imperative to conduct more studies, especially in developing and undeveloped countries, to understand this pathogen in different areas and populations.

In this study, we consecutively investigated, from July 2013 to June 2016, the epidemiological and clinical features of HMPV present in pediatric patients (≤14 years)

For peer review only - http://bmjopen.bmj.com/site/about/guidelines.xhtml

85 hospitalized with ARI in Guangzhou, southern China, using real-time PCR. The findings of this study will be helpful in understanding the distribution of HMPV in subtropical region. Our results also provide valuable insight into the clinical features of HMPV, which will be helpful in early clinical diagnosis.

For peer review only - http://bmjopen.bmj.com/site/about/guidelines.xhtml

Methods

Study design and respiratory sample collection

This was a cross-sectional study at two tertiary hospitals, the First Affiliated Hospital of Guangzhou Medical University and Sun Yat-Sen Memorial Hospital in Guangzhou. Samples in this study were taken as part of standard care. Throat swab samples (n=5133) from pediatric patients (≤14 years) hospitalized with ARI were collected at two hospitals between July 2013 and June 2016. ARI was defined as an illness that presented with at least two of the following symptoms: cough, pharyngeal discomfort, nasal obstruction, coryza, sneeze, dyspnoea, or diagnosed with pneumonia by chest radiography during the previous week. Chest radiography was performed based on the clinical situation of the patients. The samples were collected according to established clinical protocols.[17] The samples were refrigerated at 2–8°C in viral transport medium, transported on ice to the State Key Laboratory of Respiratory Diseases, and analyzed immediately or stored at -80°C before analysis as previously described.[18]

The patients' clinical presentations or diagnoses were extracted from their medical records using designed presentation cards and were retrospectively categorized into the following four groups: URTI, LRTI, systemic influenza-like symptoms, and gastrointestinal illness. Patients with nasal obstruction, coryza, sneezing, coughing, expectoration, pharyngeal discomfort, or hoarseness were categorized as having URTI. Patients with bronchiolitis, pneumonia, bronchopneumonia, increased lung markings, dyspnea, or an abnormal pulmonary

For peer review only - http://bmjopen.bmj.com/site/about/guidelines.xhtml

breath sound were categorized as having LRTI. Patients with high fever (≥38°C), chills, dizziness,, headache, myalgia, or debilitation were categorized as having systemic influenza-like symptoms. Patients with vomiting, poor appetite, or diarrhea were categorized as having gastrointestinal illness. Some patients were assigned to several clinical presentation groups. Patients with incomplete clinical data were excluded from this analysis. Increased lung markings, bronchopneumonia, pneumonia, and bronchiolitis were diagnosed by chest radiography. Abnormal pulmonary breath sounds included phlegmatic rales, wheezy rales, bubbling rales, and moist rales. Other clinical symptoms were identified by a general medical examination and clinical descriptions as previously reported.[19]

Real-time RT-PCR for HMPV detection

RNA were extracted from the throat swab samples with the QIAamp Viral RNA Mini Kit (Qiagen, Shanghai, China), according to the manufacturer's protocols. HMPV was determined by using TaqMan Real-time PCR assays, as previously reported,[18] using kits from Guangzhou HuYanSuo Medical Technology Co., Ltd according to the manufacturer's protocols. In brief, 50 µl RNA were extracted from 200 µl sample, and real-time PCR was conducted using 25 µl reaction mix containing M-MLV, Taq polymerase and 5 µl extracted RNA. Cycling conditions included an initial reverse transcription at 55°C for 10 min incubation at 94°C for 2 min, followed by 40 cycles of 94°C for 10 sec and 55°C for 35 sec (ABI-7500 real-time PCR instrument, Life Technologies, Singapore).

For peer review only - http://bmjopen.bmj.com/site/about/guidelines.xhtml

Detection of common respiratory pathogens in HMPV-positive patients

HMPV-positive samples were simultaneously tested by using TaqMan Real-time PCR assays for the following 17 respiratory pathogens : respiratory syncytial virus (RSV), parainfluenza virus types 1–4 (PIV1–4), influenza A and B viruses (InfA, InfB), adenovirus (ADV), enterovirus (EV), human coronaviruses (HCoV-229E, HCoV-OC43, HCoV-NL63, HCoV-HKU1), human rhinovirus (HRV), human bocavirus (HBoV), *Mycoplasma pneumoniae* (MP), and *Chlamydophila pneumoniae* (CP). The testing procedure was conducted using kits from Guangzhou HuYanSuo Medical Technology Co., Ltd as previously described.[18]

Statistical analysis

All statistical analyses were performed with SPSS statistical software (version 19.0; SPSS Inc., Chicago, IL, USA). To compare categorical data, the χ^2 test and Fisher's exact test were used as appropriate. All tests were two-tailed, and $p<0.05$ was considered statistically significant.

For peer review only - http://bmjopen.bmj.com/site/about/guidelines.xhtml

Results

Detection of HMPV from patients with ARI

In total, 5133 pediatric patients aging from infants to 14 years old, hospitalized with ARI between July 2013 and June 2016 were enrolled in this study. The male-to-female ratio was 1.75 (3269:1864) and the median age in months was 33 months (interquartile range, 9–48 months). Of the 5133 patients, 103 (2.0%) were positive for HMPV. The male-to-female ratio was 1.86:1 (67:36) in HMPV-positive patients and 1.75:1 (3202:1828) in HMPV-negative patients (p=0.771). The median age in months of HMPV-positive patients was 23.5 months (interquartile range, 10–36 months).

Co-detection with common respiratory pathogens in HMPV-positive patients

In this study, we also tested HMPV-positive samples for other 17 common respiratory pathogens. Of the 103 HMPV-positive patients, 84 (81.6%) patients had single HMPV detection and 19 (18.4%) patients had co-detected other respiratory pathogens. The male-to-female ratio was 2:1 (56:28) in single HMPV-positive patients and 1.38:1 (11:8) in co-pathogen-positive patients (p=0.469). Nine out of 17 respiratory pathogens (52.9%) were detected, and the most common co-detection pathogens were RSV (36.8%, 7/19), HCoV-OC43 (15.8%, 3/19), MP (15.8%, 3/19), and HBoV (15.8%, 3/19) (**Table 1**).

Age distributions of HMPV-positive patients

For peer review only - http://bmjopen.bmj.com/site/about/guidelines.xhtml

Overall, a significant difference of HMPV detection rate was found between patients >5–14 years (0.7%, 5/734) and those ≤5 years (2.2%, 98/4399) (*p*=0.004). For detail, the patients were divided into six age groups: 0–3 months, >3–6 months, >6–12 months, >1–2 years, >2–5 years, and >5–14 years. The distribution of HMPV-positive rates among these age groups was significantly different (*p*=0.03) (**Figure 1**). HMPV was most frequently detected in children >1–2 years (2.8%, 21/752). However, there was no significant difference in the frequency of HMPV detection in the following groups: 2.4% in >3–6 months (10/423), 2.2% in >6–12 months (30/1342), 2.8% in >1–2 years (21/752), and 2.3% in >2–5 years (32/1403) (*p*=0.865) (**Figure 1**). Thus, as a whole, HMPV prevalence obviously had a differential age distribution in ARI pediatric patients, predominant in patients from 3 months to 5 years old (2.4%, 93/3920)..

Seasonal distribution of HMPV

Over the 3-year study period, the prevalence of HMPV peaked twice every year (**Figure 2**). Large peaks occurred in March 2014 (8%, 16/200), May 2015 (7.6%, 8/105), and February 2016 (8.7%, 9/103). Small peaks occurred in November 2014 (1.7%, 2/121), September 2015 (2.8%, 2/72), and May 2016 (1.5%, 2/135) (**Figure 2**).

Clinical presentations of HMPV-positive patients

We analyzed the clinical presentations of 101 of 103 (98.1%) HMPV-positive patients, the other two patients were excluded from this analysis due to incomplete

For peer review only - http://bmjopen.bmj.com/site/about/guidelines.xhtml

clinical data. The main symptoms of HMPV-positive patients included cough (100.0%, 101/101), abnormal pulmonary breath sound (91.1%, 92/101), fever (88.1%, 89/101), expectoration (77.2%, 78/101), coryza (50.5%, 51/101) and wheezing (46.5%, 47/101), the main diagnosis by chest radiography was bronchopneumonia (55.4%, 56/101), followed by pneumonia (10.9%, 11/101) and bronchiolitis (7.9%, 8/101). (**Table 2**). We also compared the clinical features of single HMPV-positive patients with those of the co-pathogens-positive patients. Fever and diarrhea were two symptoms that showed statistically different between these two groups; fever (≥38˚C) was seen more common in most of the single HMPV-positive cases (91.6%,76/83) , whereas only in 72.2% (13/18) of the multi-pathogen-positive patients (*p*=0.037). Conversely, Diarrhea appeared more often in multi-pathogen-positive patients (22.2%, 4/18) than in single HMPV-positive patients (3.6%, 3/83) (*p*=0.018) (**Table 2**).

For peer review only - http://bmjopen.bmj.com/site/about/guidelines.xhtml

Discussion

Viruses are the most frequent cause of respiratory infection.[20] HMPV has been recognized as an important respiratory pathogen causing ARI since its discovery in 2001. It has been reported that HMPV was responsible for approximately 5–10% of hospitalizations of children suffering from ARI,[14] creating considerable clinical and economic burden worldwide. HMPV detection rates vary according to geographic location, and the incidence of HMPV may show seasonal or annual patterns in the same area. In the previous studies,[21-30] the HMPV detection rate was approximately 1–17% of ARI cases. In this 3-year study, 103 of 5133 (2.0%) patients were positive for HMPV, which is similar to previous reports of HMPV in patients with ARI in southern China[31] and Japan.[32]

HMPV is commonly found in the pediatric population; Children younger than 5 years of age are most susceptible to HMPV infection, and those younger than 2 years of age at the greatest risk of developing serious conditions.[15] In our study, HMPV positive cases appeared more frequently in patients ≤5 year-old (2.2%, 98/4399) than in patients >5 year-old (0.7%, 5/734) (p=0.004), which is consistent with many previous studies.[3 30 33 34] In detail, HMPV positive rate was high in children of 3 months to 5 years (2.4%, 93/3920) (**Figure 1**). This may be explained by that the immunity against HMPV passed down from mother gradually subsided as time goes on; moreover, children were more contact with outside world as they grew older. Therefore, children of this age need more attention to prevent HMPV infection. Moreover, our findings suggested no gender predominance, there was no significant

For peer review only - http://bmjopen.bmj.com/site/about/guidelines.xhtml

difference in the HMPV positive rate between males and females (p=0.771), which is consistent with previous studies.[35 36]

HMPV has a typical seasonal distribution. The activity of HMPV is largely affected by different climate factors locally. The activity of HMPV in temperate climates peaks at the end of winter or in early spring.[37 38] In this study, seasonal peaks of HMPV were detected in February 2016, March 2014, and May 2015 (**Figure 2**). This finding indicates that HMPV circulates primarily during the spring and early summer in Guangzhou, as the previous reports in other subtropical areas.[22 39] Furthermore, we also found small peaks in November 2014, September 2015, and May 2016 (**Figure 2**), the reason for this is unknown. It should be pointed out that the seasonal distribution pattern of HMPV overlaps with that of RSV in this area.[19] Knowing the epidemical characteristics of HMPV could be helpful for public health authority and medical community to lay down strategies for better controlling HMPV infection.

HMPV co-infection with other respiratory pathogens has been reported in many studies, including RSV,[40] InfA,[41] InfB,[42] PIV,[43] ADV,[43 44] HBoV,[45] HCoV ,[43 46] HRV,[43] EV,[43] MP,[41] and CP.[41] In this study, most HMPV-positive patients had single HMPV-detection (81.6%, 84/103), and there was no significant difference in the HMPV positivity rate according to sex between single HMPV-positive and co-pathogens-positive patients (p=0.469). The co-detection rate was 18.4% (19/103), and RSV (36.8%, 7/19) was the most frequently co-detected pathogen (**Table 1**), which might be due to overlapping of seasonal distribution of these two pathogens.[47]

For peer review only - http://bmjopen.bmj.com/site/about/guidelines.xhtml

Co-detection rates of HCoV-OC43, MP, HBoV, ADV, HRV, InfA, PIV2, and PIV3 were over 5%, suggesting a broad range of respiratory pathogens could co-exist with HMPV in ARI pediatric patients (**Table 1**).

HMPV most commonly causes URTI and LRTI in young children. The clinical manifestations of HMPV-positive patients are similar to those of RSV-positive patients, especially in young children,[48-50] and HMPV has been reported that the most frequent diagnoses of children hospitalized with HMPV infection were pneumonitis and bronchiolitis.[3 51 52] In some studies, bronchiolitis and recurrent wheezing/pneumonia were the main clinical diagnosis,[7 22 53 54] while in other studies, bronchopneumonia, bronchiolitis, and bronchial asthma exacerbation were the main clinical diagnosis.[34] Our study showed that 55.4% (56/101), 7.9% (8/101), and 10.9% (11/101) of HMPV-positive patients were diagnosed with bronchopneumonia, bronchiolitis, and pneumonia by chest radiography, respectively. Of all clinical presentations, cough (100.0%, 101/101), abnormal pulmonary breath sound (91.1%, 92/101), fever (88.1%, 89/101), expectoration (77.2%, 78/101), coryza (50.5%, 51/101), and wheezing (46.5%, 47/101) occurred frequently in HMPV-positive patients **(Table 2)**, similar to those reported previously.[16 29 52 55]

Most of the HMPV-positive patients had a fever (88.1%, 89/101); it appeared in the single HMPV-positive patients more frequently than in the co-pathogens-positive patients(p=0.037) (**Table 2**). Although diarrhea was not a major symptom in HMPV-positive patients (7.0%, 7/101), but it had a higher incidence rate in co-pathogen-positive patients than in single HMPV-positive patients (p=0.018),

For peer review only - http://bmjopen.bmj.com/site/about/guidelines.xhtml

suggesting that diarrhea could be caused by other pathogens. No significant difference was found in the other clinical presentations between single HMPV-positive and co-pathogens-positive patients, similar to the results of a previous study.[51]

In this study, RSV (36.8%, 7/19) was the most frequently co-detected pathogen in HMPV-positive patients (**Table 1**). Whether HMPV/RSV co-infection is more severe than the respective mono-infections remains unclear. A previous study showed that children with HMPV/RSV co-infection were more likely to develop pneumonia; however, disease severity was not increased.[53] Conversely, Semple et al.[56] reported that HMPV/RSV co-infection can cause more severe bronchiolitis in patients. In our study, no significant difference in the diagnosis of pneumonia was observed between single HMPV-positive and co-pathogens-positive patients. It has been confirmed that a history of prematurity, particular age groups, and the presence of chronic diseases increases the risk of severe LRTI among HMPV- and RSV-infected children.[51] Our study also suggested that the clinical manifestations of HMPV infection are complex and diverse; the data might be helpful in the diagnosis of HMPV.

The study had some limitations. Firstly, collection of symptoms and physical findings in infants and young children often requires the experience of medical staff, patient's cooperation, and the knowledge of patient's guardian; but it is still possible to have incomplete or even false description of patients' manifestations. Secondly, because our study mainly focused on HMPV, other common respiratory pathogens including bacterial pathogens were not tested in all samples, and accordingly, our study could not give a fully picture of respiratory pathogen infection in hospitalized

For peer review only - http://bmjopen.bmj.com/site/about/guidelines.xhtml

293 pediatric patients with ARI. Thirdly, the characteristics of HMPV-negative patients were not further analyzed because our study only focused on the epidemical and clinical features of HMPV positive cases. That might affect our understanding of the clinical feature of HMPV-positive patients. Despite these shortcomings, our results provide valuable insight into the epidemiological and clinical characteristics of HMPV present in patients hospitalized with acute respiratory illness (ARI) in a subtropical region, Guangzhou, southern China.

For peer review only

For peer review only - http://bmjopen.bmj.com/site/about/guidelines.xhtml

Conclusions

HMPV is a common respiratory pathogen in children with ARI in Guangzhou , China, particularly in children from 3 month to 5 years old. HMPV epidemic has a seasonal variation. The clinical characteristics of HMPV infection has its own pattern in children aged from infants to juveniles. Bronchopneumonia is the major clinical diagnosis. In the future, our data should be taken into account when local public health authority and medical community try to manage HMPV infection in children.

For peer review only

Acknowledgements

We thank the study volunteers for their generous participation. We thank Yinghua Zhou, Haiping Huang, Jing Zhang, and Jing Ma for technical assistance. We thank Professor Xiaofeng Li and Dr. Zhengshi Lin for paper polishing.

Contributors

RZ, WKL, LZ and DLL designed the study. LZ, DLL, WKL, SYQ, DX, XL and LTT performed pathogen testing. DHC and WPT collected clinical data. All authors participated in the data analysis. LZ, DLL, RZ and WKL drafted the manuscript. All authors read and approved the final version of this manuscript.

Funding

This study was supported by The State Major Infectious Disease Research Program (RZ) (2017ZX10103011-003), National Natural Science Foundation of China (WKL) (31500143), Guangzhou Science and Technology Program key projects (RZ) (201508020252). The funders had no role in study design, data collection and analysis, decision to publish, or preparation of the manuscript.

Competing interests

None declared.

Ethics approval

For peer review only

For peer review only - http://bmjopen.bmj.com/site/about/guidelines.xhtml

The study was approved by The First Affiliated Hospital of Guangzhou Medical University Ethics Committee. Informed written consent was obtained from parents or legal guardians.

Data sharing statement

No additional data are available.

For peer review only - http://bmjopen.bmj.com/site/about/guidelines.xhtml

REFERENCES

1. van den Hoogen BG, Herfst S, Sprong L, et al. Antigenic and genetic variability of human metapneumoviruses. *Emerg Infect Dis* 2004;10(4):658-66. doi: 10.3201/eid1004.030393
2. van den Hoogen BG, de Jong JC, Groen J, et al. A newly discovered human pneumovirus isolated from young children with respiratory tract disease. *Nat Med* 2001;7(6):719-24. doi: 10.1038/89098
3. Boivin G, Abed Y, Pelletier G, et al. Virological features and clinical manifestations associated with human metapneumovirus: a new paramyxovirus responsible for acute respiratory-tract infections in all age groups. *J Infect Dis* 2002;186(9):1330-4. doi: 10.1086/344319
4. Falsey AR, Walsh EE. Viral pneumonia in older adults. *Clin Infect Dis* 2006;42(4):518-24. doi: 10.1086/499955
5. Kahn JS. Epidemiology of human metapneumovirus. *Clin Microbiol Rev* 2006;19(3):546-57. doi: 10.1128/CMR.00014-06
6. Davis CR, Stockmann C, Pavia AT, et al. Incidence, Morbidity, and Costs of Human Metapneumovirus Infection in Hospitalized Children. *J Pediatric Infect Dis Soc* 2016;5(3):303-11. doi: 10.1093/jpids/piv027
7. Edwards KM, Zhu Y, Griffin MR, et al. Burden of human metapneumovirus infection in young children. *N Engl J Med* 2013;368(7):633-43. doi: 10.1056/NEJMoa1204630
8. Foulongne V, Guyon G, Rodiere M, et al. Human metapneumovirus infection in young children hospitalized with respiratory tract disease. *Pediatr Infect Dis J* 2006;25(4):354-9. doi: 10.1097/01.inf.0000207480.55201.f6
9. Heikkinen T, Osterback R, Peltola V, et al. Human metapneumovirus infections in children. *Emerg Infect Dis* 2008;14(1):101-6. doi: 10.3201/eid1401.070251
10. Nicholson KG, McNally T, Silverman M, et al. Rates of hospitalisation for influenza, respiratory syncytial virus and human metapneumovirus among infants and young children. *Vaccine* 2006;24(1):102-8. doi: 10.1016/j.vaccine.2005.02.004
11. Sloots TP, Mackay IM, Bialasiewicz S, et al. Human metapneumovirus, Australia, 2001-2004. *Emerg Infect Dis* 2006;12(8):1263-6. doi: 10.3201/eid1208.051239
12. Williams JV, Edwards KM, Weinberg GA, et al. Population-based incidence of human metapneumovirus infection among hospitalized children. *J Infect Dis* 2010;201(12):1890-8. doi: 10.1086/652782
13. Ebihara T, Endo R, Kikuta H, et al. Seroprevalence of human metapneumovirus in Japan. *J Med Virol* 2003;70(2):281-3. doi: 10.1002/jmv.10391
14. Panda S, Mohakud NK, Pena L, et al. Human metapneumovirus: review of an important respiratory pathogen. *Int J Infect Dis* 2014;25:45-52. doi: 10.1016/j.ijid.2014.03.1394
15. Broor S, Bharaj P, Chahar HS. Human metapneumovirus: a new respiratory pathogen. *J Biosci* 2008;33(4):483-93.
16. Jain B, Singh AK, Dangi T, et al. High prevalence of human metapneumovirus subtype B in cases presenting as severe acute respiratory illness: an experience at tertiary care hospital. *Clin Respir J* 2014;8(2):225-33. doi: 10.1111/crj.12064
17. Liu WK, Liu Q, Chen DH, et al. Epidemiology and clinical presentation of the four human parainfluenza virus types. *BMC Infect Dis* 2013;13:28. doi: 10.1186/1471-2334-13-28
18. Liu WK, Chen DH, Liu Q, et al. Detection of human bocavirus from children and adults with acute respiratory tract illness in Guangzhou, southern China. *BMC Infect Dis* 2011;11:345. doi:

For peer review only - http://bmjopen.bmj.com/site/about/guidelines.xhtml

10.1186/1471-2334-11-345

19. Liu W, Chen D, Tan W, et al. Epidemiology and Clinical Presentations of Respiratory Syncytial Virus Subgroups A and B Detected with Multiplex Real-Time PCR. *PLoS One* 2016;11(10):e0165108.DOI: 10.1371/journal.pone.08. doi: 10.1371/journal.pone.0165108
20. Williams BG, Gouws E, Boschi-Pinto C, et al. Estimates of world-wide distribution of child deaths from acute respiratory infections. *Lancet Infect Dis* 2002;2(1):25-32.
21. Escobar C, Luchsinger V, de Oliveira DB, et al. Genetic variability of human metapneumovirus isolated from Chilean children, 2003-2004. *J Med Virol* 2009;81(2):340-4. doi: 10.1002/jmv.21399
22. Garcia-Garcia ML, Calvo C, Rey C, et al. Human metapnuemovirus infections in hospitalized children and comparison with other respiratory viruses. 2005-2014 prospective study. *PLoS One* 2017;12(3):e0173504.DOI: 10.1371/journal.pone.04. doi: 10.1371/journal.pone.0173504
23. Ljubin-Sternak S, Santak M, Cepin-Bogovic J, et al. Detection of genetic lineages of human metapneumovirus in Croatia during the winter season 2005/2006. *J Med Virol* 2008;80(7):1282-7. doi: 10.1002/jmv.21196
24. McCracken JP, Arvelo W, Ortiz J, et al. Comparative epidemiology of human metapneumovirus- and respiratory syncytial virus-associated hospitalizations in Guatemala. *Influenza Other Respir Viruses* 2014;8(4):414-21. doi: 10.1111/irv.12251
25. Nor'e SS, Sam IC, Mohamad Fakri EF, et al. Phylogenetic analysis of human metapneumovirus among children with acute respiratory infections in Kuala Lumpur, Malaysia. *Trop Biomed* 2014;31(3):562-6.
26. Ordas J, Boga JA, Alvarez-Arguelles M, et al. Role of metapneumovirus in viral respiratory infections in young children. *J Clin Microbiol* 2006;44(8):2739-42. doi: 10.1128/JCM.00164-06
27. Rafiefard F, Yun Z, Orvell C. Epidemiologic characteristics and seasonal distribution of human metapneumovirus infections in five epidemic seasons in Stockholm, Sweden, 2002-2006. *J Med Virol* 2008;80(9):1631-8. doi: 10.1002/jmv.21242
28. Rodriguez PE, Adamo MP, Paglini MG, et al. [Monoinfection of human Metapneumovirus in Cordoba: first clinical and epidemiological research in children with respiratory infection in 2011]. *Rev Fac Cien Med Univ Nac Cordoba* 2016;73(3):170-75.
29. Zeng SZ, Xiao NG, Zhong LL, et al. Clinical features of human metapneumovirus genotypes in children with acute lower respiratory tract infection in Changsha, China. *J Med Virol* 2015;87(11):1839-45. doi: 10.1002/jmv.24249
30. Zhu RN, Qian Y, Zhao LQ, et al. Characterization of human metapneumovirus from pediatric patients with acute respiratory infections in a 4-year period in Beijing, China. *Chin Med J (Engl)* 2011;124(11):1623-8.
31. Cai XY, Wang Q, Lin GY, et al. Respiratory virus infections among children in South China. *J Med Virol* 2014;86(7):1249-55. doi: 10.1002/jmv.23931
32. Matsuzaki Y, Itagaki T, Abiko C, et al. Clinical impact of human metapneumovirus genotypes and genotype-specific seroprevalence in Yamagata, Japan. *J Med Virol* 2008;80(6):1084-9. doi: 10.1002/jmv.21194
33. Barbosa Ramirez J, Pulido Dominguez P, Rey Benito G, et al. Human respiratory syncytial virus and metapneumovirus in patients with acute respiratory infection in Colombia, 2000 - 2011. *Rev Panam Salud Publica* 2014;36(2):101-9.

For peer review only - http://bmjopen.bmj.com/site/about/guidelines.xhtml

34. Zhang C, Du LN, Zhang ZY, et al. Detection and genetic diversity of human metapneumovirus in hospitalized children with acute respiratory infections in Southwest China. *J Clin Microbiol* 2012;50(8):2714-9. doi: 10.1128/JCM.00809-12

35. McAdam AJ, Hasenbein ME, Feldman HA, et al. Human metapneumovirus in children tested at a tertiary-care hospital. *J Infect Dis* 2004;190(1):20-6. doi: 10.1086/421120

36. Boivin G, De Serres G, Cote S, et al. Human metapneumovirus infections in hospitalized children. *Emerg Infect Dis* 2003;9(6):634-40. doi: 10.3201/eid0906.030017

37. Maggi F, Pifferi M, Vatteroni M, et al. Human metapneumovirus associated with respiratory tract infections in a 3-year study of nasal swabs from infants in Italy. *J Clin Microbiol* 2003;41(7):2987-91.

38. van den Hoogen BG, van Doornum GJ, Fockens JC, et al. Prevalence and clinical symptoms of human metapneumovirus infection in hospitalized patients. *J Infect Dis* 2003;188(10):1571-7. doi: 10.1086/379200

39. Peiris JS, Tang WH, Chan KH, et al. Children with respiratory disease associated with metapneumovirus in Hong Kong. *Emerg Infect Dis* 2003;9(6):628-33. doi: 10.3201/eid0906.030009

40. Greensill J, McNamara PS, Dove W, et al. Human metapneumovirus in severe respiratory syncytial virus bronchiolitis. *Emerg Infect Dis* 2003;9(3):372-5. doi: 10.3201/eid0903.020289

41. Lin PY, Lin TY, Huang YC, et al. Human metapneumovirus and community-acquired pneumonia in children. *Chang Gung Med J* 2005;28(10):683-8.

42. Ghattas C, Mossad SB. Fatal human metapneumovirus and influenza B virus coinfection in an allogeneic hematopoietic stem cell transplant recipient. *Transpl Infect Dis* 2012;14(5):E41-3.DOI: 10.1111/j.399-3062.2012.00766.x. doi: 10.1111/j.1399-3062.2012.00766.x

43. Fathima S, Lee BE, May-Hadford J, et al. Use of an innovative web-based laboratory surveillance platform to analyze mixed infections between human metapneumovirus (hMPV) and other respiratory viruses circulating in Alberta (AB), Canada (2009-2012). *Viruses* 2012;4(11):2754-65. doi: 10.3390/v4112754

44. Kaida A, Kubo H, Goto K, et al. Co-infection of human metapneumovirus with adenovirus or respiratory syncytial virus among children in Japan. *Microbiol Immunol* 2007;51(7):679-83.

45. Pilger DA, Cantarelli VV, Amantea SL, et al. Detection of human bocavirus and human metapneumovirus by real-time PCR from patients with respiratory symptoms in Southern Brazil. *Mem Inst Oswaldo Cruz* 2011;106(1):56-60.

46. Esposito S, Daleno C, Prunotto G, et al. Impact of viral infections in children with community-acquired pneumonia: results of a study of 17 respiratory viruses. *Influenza Other Respir Viruses* 2013;7(1):18-26. doi: 10.1111/j.1750-2659.2012.00340.x

47. Kahn JS. Newly discovered respiratory viruses: significance and implications. *Curr Opin Pharmacol* 2007;7(5):478-83. doi: 10.1016/j.coph.2007.07.004

48. Wolf DG, Greenberg D, Kalkstein D, et al. Comparison of human metapneumovirus, respiratory syncytial virus and influenza A virus lower respiratory tract infections in hospitalized young children. *Pediatr Infect Dis J* 2006;25(4):320-4. doi: 10.1097/01.inf.0000207395.80657.cf

49. Wilkesmann A, Schildgen O, Eis-Hubinger AM, et al. Human metapneumovirus infections cause similar symptoms and clinical severity as respiratory syncytial virus infections. *Eur J*

For peer review only - http://bmjopen.bmj.com/site/about/guidelines.xhtml

Pediatr 2006;165(7):467-75. doi: 10.1007/s00431-006-0105-4

50. Adams O, Weis J, Jasinska K, et al. Comparison of human metapneumovirus, respiratory syncytial virus and Rhinovirus respiratory tract infections in young children admitted to hospital. *J Med Virol* 2015;87(2):275-80. doi: 10.1002/jmv.24025

51. Moe N, Krokstad S, Stenseng IH, et al. Comparing Human Metapneumovirus and Respiratory Syncytial Virus: Viral Co-Detections, Genotypes and Risk Factors for Severe Disease. *PLoS One* 2017;12(1):e0170200.DOI: 10.1371/journal.pone.00. doi: 10.1371/journal.pone.0170200

52. Yan XL, Li YN, Tang YJ, et al. Clinical characteristics and viral load of respiratory syncytial virus and human metapneumovirus in children hospitaled for acute lower respiratory tract infection. *J Med Virol* 2017;89(4):589-97. doi: 10.1002/jmv.24687

53. Caracciolo S, Minini C, Colombrita D, et al. Human metapneumovirus infection in young children hospitalized with acute respiratory tract disease: virologic and clinical features. *Pediatr Infect Dis J* 2008;27(5):406-12. doi: 10.1097/INF.0b013e318162a164

54. Williams JV, Harris PA, Tollefson SJ, et al. Human metapneumovirus and lower respiratory tract disease in otherwise healthy infants and children. *N Engl J Med* 2004;350(5):443-50. doi: 10.1056/NEJMoa025472

55. Schuster JE, Khuri-Bulos N, Faouri S, et al. Human Metapneumovirus Infection in Jordanian Children: Epidemiology and Risk Factors for Severe Disease. *Pediatr Infect Dis J* 2015;34(12):1335-41. doi: 10.1097/INF.0000000000000892

56. Semple MG, Cowell A, Dove W, et al. Dual infection of infants by human metapneumovirus and human respiratory syncytial virus is strongly associated with severe bronchiolitis. *J Infect Dis* 2005;191(3):382-6. doi: 10.1086/426457

For peer review only

For peer review only - http://bmjopen.bmj.com/site/about/guidelines.xhtml

Figure legends

Figure 1 Age distributions of patients with HMPV.

[a]Date were presented as HMPV positive rate (number of HMPV-positive patients/number of patients in each age group); m: month(s); y: year(s).

Figure 2 Seasonal distribution of HMPV infection in pediatric patients hospitalized with acute respiratory infection from July 2013 to June 2016 in Guangzhou.

For peer review only

For peer review only - http://bmjopen.bmj.com/site/about/guidelines.xhtml

500 **Table 1. Distribution of co-pathogens in 19 HMPV-positive patients**

Co-pathogens[a]	Patient No.
RSV/HMPV	4
HCoV-OC43/HMPV	3
ADV/HMPV	2
HRV/HMPV	2
MP/HMPV	1
PIV2/HMPV	1
PIV3/HMPV	1
RSV/MP/HMPV	1
RSV/InfA/HMPV	1
RSV/HBoV/HMPV	1
MP/HBoV/HMPV	1
InfA/HBoV/HMPV	1

501 [a]HMPV-positive patients were tested for 17 common respiratory pathogens.

502 InfB, PIV1, PIV4, EV, HCoV-HKU1, HCoV-229E, HCoV-NL63, CP were not detected.

503

504

For peer review only - http://bmjopen.bmj.com/site/about/guidelines.xhtml

Table 2. Clinical presentations in HMPV-positive patients

Diagnosis/Symptom	Total HMPV (n=101)	Patients with single HMPV and co-pathogens		
		Single HMPV (n = 83)	Co-pathogens (n= 18)	p value[b]
**URTI**				
Nasal obstruction	40(39.6)	34(41.0)	6(33.3)	0.605
Coryza	51(50.5)	44(53.0)	7(38.9)	0.309
Sneeze	1(1.0)	1(1.2)	0(0)	–[c]
Cough	101(100)	83(100)	18(100)	–[d]
Expectoration	78(77.2)	66(79.5)	12(66.7)	0.351
Pharyngeal discomfort	2(2.0)	2(2.4)	0(0)	–[c]
Hoarseness	1(1.0)	1(1.2)	0(0)	–[c]
**LRTI**				
Abnormal pulmonary breath sound [a]	92(91.1)	75(90.4)	17(94.4)	0.582
Increasing lung markings	6(6.0)	4(4.8)	2(11.1)	0.29
wheezing	47(46.5)	40(48.2)	7(38.9)	0.473
anhelation	24(23.8)	21(25.3)	3(16.7)	0.551
Bronchiolitis	8(7.9)	7(8.4)	1(5.6)	0.682
Pneumonia	11(10.9)	10(12.1)	1(5.6)	0.684
bronchopneumonia	56(55.4)	46(55.4)	10(55.6)	0.992
**Systemic influenza-like symptoms**				
Fever (≥38˚C)	89(88.1)	76(91.6)	13(72.2)	**0.037**
Chill	7(6.9)	6(7.2)	1(5.6)	0.8
Debilitation	11(10.9)	11(13.3)	0(0)	–[c]
**Gastrointestinal illness**				
Vomiting	21(20.8)	16(19.3)	5(27.8)	0.522
Poor appetite	19(18.8)	17(20.5)	2(11.1)	0.513
Diarrhea	7(7.0)	3(3.6)	4(22.2)	**0.018**

Data are presented as No. (%) of each group.

Percentages sum to >100% because some patients had >1 clinical presentations.

[a]Including phlegmatic rales, wheezing rales, bubbling rales, and moist rales.

[b]Two-tailed χ^2 test comparing the distribution of each illness or diagnosis between HMPV mono- and co-infected patients.

[c]Not tested because the number of positive samples obtained was small.

[d]Not tested because the rate of positive samples obtained was 100%.

For peer review only - http://bmjopen.bmj.com/site/about/guidelines.xhtml

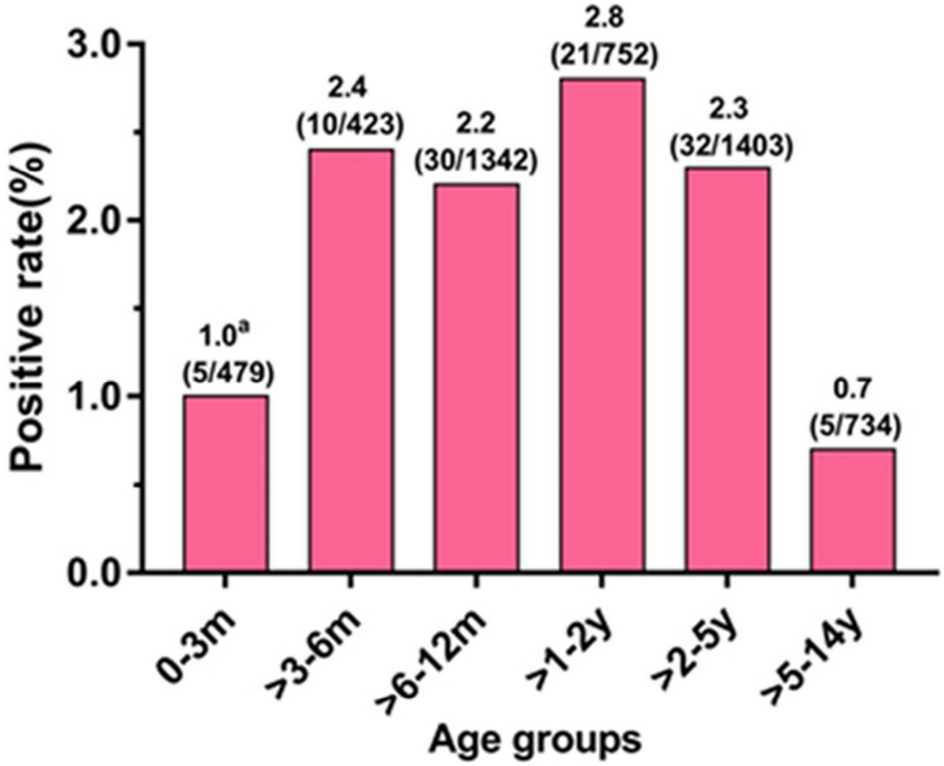

Figure 1 Age distributions of patients with HMPV.
aDate were presented as HMPV positive rate (number of HMPV-positive patients/number of patients in each age group); m: month(s); y: year(s).

42x35mm (300 x 300 DPI)

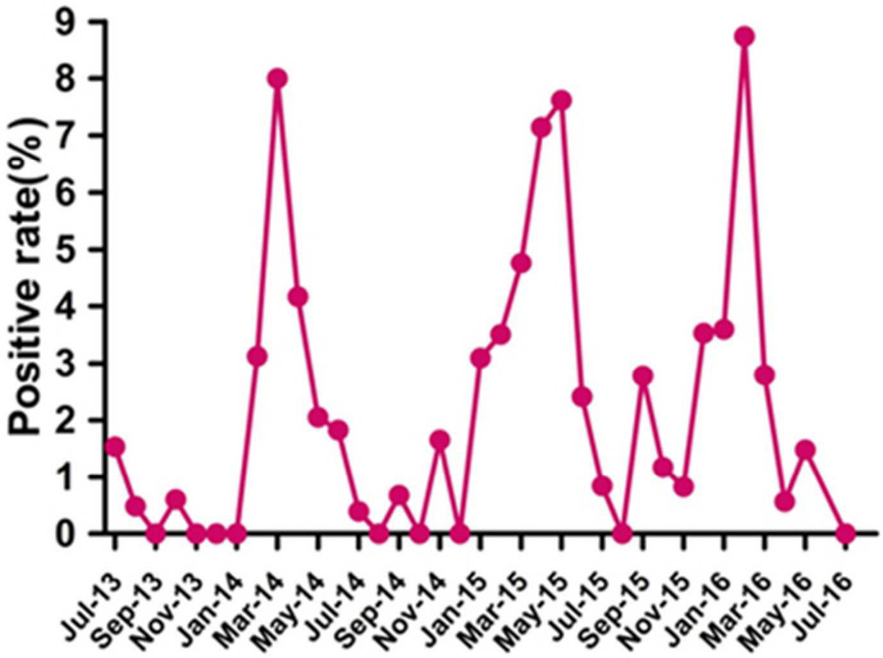

Figure 2 Seasonal distribution of HMPV infection in pediatric patients hospitalized with acute respiratory infection from July 2013 to June 2016 in Guangzhou.

39x31mm (300 x 300 DPI)

STROBE 2007 (v4)Statement—Checklist of items that should be included in reports of *cross-sectional studies*

Section/Topic	Item #	Recommendation	Reported on page #
**Title and abstract**	1	(*a*) Indicate the study's design with a commonly used term in the title or the abstract	1
		(*b*) Provide in the abstract an informative and balanced summary of what was done and what was found	2-3
**Introduction**			
Background/rationale	2	Explain the scientific background and rationale for the investigation being reported	4
Objectives	3	State specific objectives, including any prespecified hypotheses	4-5
**Methods**			
Study design	4	Present key elements of study design early in the paper	6
Setting	5	Describe the setting, locations, and relevant dates, including periods of recruitment, exposure, follow-up, and data collection	6
Participants	6	(*a*) Give the eligibility criteria, and the sources and methods of selection of participants	6
Variables	7	Clearly define all outcomes, exposures, predictors, potential confounders, and effect modifiers. Give diagnostic criteria, if applicable	6-7
Data sources/ measurement	8*	For each variable of interest, give sources of data and details of methods of assessment (measurement). Describe comparability of assessment methods if there is more than one group	7
Bias	9	Describe any efforts to address potential sources of bias	*6*
Study size	10	Explain how the study size was arrived at	6
Quantitative variables	11	Explain how quantitative variables were handled in the analyses. If applicable, describe which groupings were chosen and why	7-8
Statistical methods	12	(*a*) Describe all statistical methods, including those used to control for confounding	8
		(*b*) Describe any methods used to examine subgroups and interactions	8
		(*c*) Explain how missing data were addressed	7
		(*d*) If applicable, describe analytical methods taking account of sampling strategy	8
		(*e*) Describe any sensitivity analyses	
**Results**			

For peer review only

Participants	13*	(a) Report numbers of individuals at each stage of study—eg numbers potentially eligible, examined for eligibility, confirmed eligible, included in the study, completing follow-up, and analysed	9
		(b) Give reasons for non-participation at each stage	10
		(c) Consider use of a flow diagram	
Descriptive data	14*	(a) Give characteristics of study participants (eg demographic, clinical, social) and information on exposures and potential confounders	9-11
		(b) Indicate number of participants with missing data for each variable of interest	9
Outcome data	15*	Report numbers of outcome events or summary measures	9-11
Main results	16	(*a*) Give unadjusted estimates and, if applicable, confounder-adjusted estimates and their precision (eg, 95% confidence interval). Make clear which confounders were adjusted for and why they were included	9-11
		(*b*) Report category boundaries when continuous variables were categorized	9-10
		(*c*) If relevant, consider translating estimates of relative risk into absolute risk for a meaningful time period	
Other analyses	17	Report other analyses done—eg analyses of subgroups and interactions, and sensitivity analyses	
**Discussion**			
Key results	18	Summarise key results with reference to study objectives	12-15
Limitations	19	Discuss limitations of the study, taking into account sources of potential bias or imprecision. Discuss both direction and magnitude of any potential bias	15-16
Interpretation	20	Give a cautious overall interpretation of results considering objectives, limitations, multiplicity of analyses, results from similar studies, and other relevant evidence	12-16
Generalisability	21	Discuss the generalisability (external validity) of the study results	12-16
**Other information**			
Funding	22	Give the source of funding and the role of the funders for the present study and, if applicable, for the original study on which the present article is based	18

*Give information separately for cases and controls in case-control studies and, if applicable, for exposed and unexposed groups in cohort and cross-sectional studies.

Note: An Explanation and Elaboration article discusses each checklist item and gives methodological background and published examples of transparent reporting. The STROBE checklist is best used in conjunction with this article (freely available on the Web sites of PLoS Medicine at http://www.plosmedicine.org/, Annals of Internal Medicine at http://www.annals.org/, and Epidemiology at http://www.epidem.com/). Information on the STROBE Initiative is available at www.strobe-statement.org.

For peer review only

BMJ Open

Epidemiology and clinical features of human metapneumovirus in hospitalized pediatric patients with acute respiratory illness: a cross-sectional study in southern China, from 2013 to 2016

Journal:	*BMJ Open*
Manuscript ID	bmjopen-2017-019308.R2
Article Type:	Research
Date Submitted by the Author:	22-Nov-2017

Complete List of Authors:	Zhang, Ling; State Key Laboratory of Respiratory Diseases, The First Affiliated Hospital of Guangzhou Medical University, Guangzhou Medical University<br>Liu, Donglan; State Key Laboratory of Respiratory Diseases, The First Affiliated Hospital of Guangzhou Medical University, Guangzhou Medical University<br>Chen, Dehui; Department of Pediatrics, The First Affiliated Hospital of Guangzhou Medical University, Guangzhou Medical University<br>Tan, Weiping ; Sun Yat-Sen Memorial Hospital, Sun Yat-Sen University<br>Qiu, Shuyan; State Key Laboratory of Respiratory Diseases, The First Affiliated Hospital of Guangzhou Medical University, Guangzhou Medical University<br>Xu, Duo; State Key Laboratory of Respiratory Diseases, The First Affiliated Hospital of Guangzhou Medical University, Guangzhou Medical University<br>Li, Xiao; State Key Laboratory of Respiratory Diseases, The First Affiliated Hospital of Guangzhou Medical University, Guangzhou Medical University<br>Liu, Tiantian; State Key Laboratory of Respiratory Diseases, The First Affiliated Hospital of Guangzhou Medical University, Guangzhou Medical University<br>Liu, Wenkuan; State Key Laboratory of Respiratory Diseases, The First Affiliated Hospital of Guangzhou Medical University, Guangzhou Medical University<br>Zhou, Rong; State Key Laboratory of Respiratory Diseases, The First Affiliated Hospital of Guangzhou Medical University, Guangzhou Medical University

**Primary Subject Heading**:	Infectious diseases
Secondary Subject Heading:	Epidemiology, Paediatrics, Infectious diseases
Keywords:	Human metapneumovirus, Epidemiology < TROPICAL MEDICINE, Clinical feature, Acute respiratory illness

For peer review only

Epidemiology and clinical features of human metapneumovirus in hospitalized pediatric patients with acute respiratory illness: a cross-sectional study in southern China, from 2013 to 2016

Ling Zhang[1†], Donglan Liu[1†], Dehui Chen[2], Weiping Tan[3], Shuyan Qiu[1], Duo Xu[1], Xiao Li[1], Tiantian Liu[1], Wenkuan Liu[1]*, Rong Zhou[1]*

[1]State Key Laboratory of Respiratory Diseases, The First Affiliated Hospital of Guangzhou Medical University, Guangzhou Medical University, Guangzhou, People's Republic of China.

[2]Department of Pediatrics, The First Affiliated Hospital of Guangzhou Medical University, Guangzhou Medical University, Guangzhou, People's Republic of China.

[3]Sun Yat-Sen Memorial Hospital, Sun Yat-Sen University, Guangzhou, People's Republic of China.

*Correspondence to: Wenkuan Liu; ahlwk2000-2004@163.com and Rong Zhou; zhourong@gird.cn

[†]Ling Zhang and Donglan Liu contributed equally to this work.

For peer review only - http://bmjopen.bmj.com/site/about/guidelines.xhtml

Abstract

Objectives: Human metapneumovirus (HMPV) is one of the most important respiratory viral pathogens affecting infants and children worldwide. Our study was describes the epidemiological and clinical characteristics of HMPV present in patients hospitalized with acute respiratory illness (ARI) in Guangzhou, southern China.

Study design: A cross-sectional study.

Setting: Two tertiary hospitals in Guangzhou.

Participants and methods: Throat swabs were collected over a 3-year period from 5133 pediatric patients (≤14 years) hospitalized with ARI. HMPV-positive patients' clinical presentations (101/103) were recorded for further analysis.

Results: Of the 5133 patients included in the study, 103 (2.0%) were positive for HMPV. HMPV was more prevalent in children ≤5 years (2.2%, 98/4399) compared with older children (>5–14 years) (0.7%, 5/734) (p=0.004). Two seasonal HMPV peaks were observed each year and mainly occurred in spring and early summer. Overall, 18.4% (19/103) of HMPV-positive patients were co-detected with other pathogens, most frequently respiratory syncytial virus (36.8%, 7/19). HMPV-positive patients presented with a wide spectrum of clinical features, including cough (100.0%, 101/101), abnormal pulmonary breath sound (91.1%, 92/101), fever (88.1%, 89/101), expectoration (77.2%, 78/101), coryza (50.5%, 51/101) and wheezing (46.5%, 47/101). The main diagnosis of HMPV-positive patients was bronchopneumonia (55.4%, 56/101). Fever (≥38˚C) (91.6%, 76/83) was detected more often in patients with only HMPV detected than in patients with HMPV plus other pathogen(s) detected

For peer review only - http://bmjopen.bmj.com/site/about/guidelines.xhtml

(72.2%, 13/18) (p=0.037), whereas diarrhea was more common in patients with HMPV plus other pathogen(s) detected (22.2%, 4/18), compared with patients with HMPV only (3.6%, 3/83) (p=0.018).

Conclusions: HMPV is an important respiratory pathogen in children with ARI in Guangzhou, particularly in children ≤5 years old. HMPV has a seasonal variation. Bronchopneumonia is a major diagnosis in HMPV-positive patients.

Strengths and limitations of this study

- 5133 patients hospitalized with acute respiratory illness at two large municipal tertiary hospitals (hospital beds ＞1000) were enrolled on the study over 3 years.
- Patients aged from 1 day to 14 years old were tested for HMPV using Taqman real-time PCR.
- Clinical characteristics of patients with HMPV-positive were recorded.
- There was incomplete HMPV co-pathogen detection, because bacterial pathogens were not detected for HMPV-positive patients.
- The clinical characteristics of HMPV-negative patients and outcome data (discharge/death) for the HMPV-positive patients were not collected for further analysis, limiting our understanding of this pathogen.

Introduction

Human metapneumovirus (HMPV) is a nonsegmented, negative-sense single-stranded RNA virus, which belongs to the *Paramyxoviridae* family.[1] HMPV was

For peer review only - http://bmjopen.bmj.com/site/about/guidelines.xhtml

first discovered in 2001 in the Netherlands, after being isolated from a pediatric patient with acute respiratory illness (ARI).[2] Since then, HMPV has been associated with acute respiratory disease in individuals of all ages worldwide. Children, elderly and immunocompromised adults are most at risk of contracting the virus.[3-5] Children younger than 5 years of age seem to be particularly susceptible to HMPV. Previous studies,[6-12] which were mainly conducted in developed countries, have revealed that approximately 2.5%–11.3% of respiratory samples were positive for HMPV in children ≤5 years, and 90% of individuals were seropositive for HMPV by 5 years of age.[13]

HMPV causes a variety of clinical symptoms ranging from a mild upper respiratory tract infection (URTI) to life-threatening lower respiratory tract infection (LRTI).[3 14-16] However, to date, there is no effective vaccine or specific medication either for prevention or treatment of HMPV infection. Consequently, it is imperative to conduct more studies, especially in low and middle-income countries, to understand this pathogen in different areas and populations.

In this study, we investigated the epidemiological and clinical features of HMPV in pediatric patients, from July 2013 to June 2016. The findings of this study will help understand the distribution of HMPV in a subtropical region. Our results also provide a valuable insight into the clinical features of HMPV, which will improve early clinical diagnosis.

For peer review only - http://bmjopen.bmj.com/site/about/guidelines.xhtml

Methods

Study design and respiratory sample collection

We conducted a cross-sectional study at two tertiary hospitals in Guangzhou, southern China. Throat swab samples (n=5133) from pediatric patients (≤14 years) hospitalized with ARI, were collected at two hospitals between July 2013 and June 2016. ARI was defined as an illness that presented with at least two of the following clinical presentations: cough, nasal obstruction, coryza, sneeze, dyspnoea during the previous week. Patients, who were diagnosed with pneumonia by chest radiography during the previous week, were also included in the study, even if they did not show the clinical features described above. Some patients, who had been cured and discharged some time ago and readmitted because of new episodes of ARI, if met the recruitment criteria, were included in the study as new cases, otherwise excluded. Chest radiography was performed based on the clinical situation of the patients. The samples were collected according to established clinical protocols.[17] The samples were refrigerated at 2–8°C in viral transport medium, transported on ice to the State Key Laboratory of Respiratory Diseases, and analyzed immediately or stored at -80°C before analysis as previously described.[18]

The patients' clinical presentations or diagnoses were recorded from patients' medical records by attending physicians, using designed presentation cards and were categorized retrospectively into the following four groups: URTI, LRTI, systemic influenza-like symptoms, and gastrointestinal illness. Patients with nasal obstruction, coryza, sneezing, coughing, expectoration, or hoarseness were categorized as

For peer review only - http://bmjopen.bmj.com/site/about/guidelines.xhtml

having URTI. Patients with bronchiolitis, pneumonia, bronchopneumonia, increased lung markings, dyspnea, or an abnormal pulmonary breath sound were categorized as having LRTI. Patients with a high fever (≥38°C), chills, or debilitation were categorized as having systemic influenza-like symptoms. Patients with vomiting, poor appetite, or diarrhea were categorized as having gastrointestinal illness. Some patients were assigned to several clinical presentation groups. Patients with incomplete clinical data were excluded from this analysis. Increased lung markings, bronchopneumonia, pneumonia, and bronchiolitis were diagnosed by chest radiography. Abnormal pulmonary breath sounds included phlegmatic, wheezy, bubbling, and moist rales. Other clinical symptoms were identified by a general medical examination and clinical descriptions, as previously reported.[19]

Real-time PCR for HMPV detection

RNA was extracted from the throat swab samples with the QIAamp Viral RNA Mini Kit (Qiagen, Shanghai, China), according to the manufacturer's protocols. HMPV was identified by using TaqMan Real-time PCR assays, as previously reported,[18] using kits from Guangzhou HuYanSuo Medical Technology Co., Ltd according to the manufacturer's protocols. In brief, 50 μl RNA were extracted from a 200 μl sample, and real-time PCR was conducted using 25 μl reaction mix, containing M-MLV, Taq polymerase and 5 μl extracted RNA. Cycling conditions included an initial reverse transcription at 55°C for 10 min, incubation at 94°C for 2 min, followed by 40 cycles of 94°C for 10 sec and 55°C for 35 sec (ABI-7500 real-time PCR instrument, Life

For peer review only - http://bmjopen.bmj.com/site/about/guidelines.xhtml

Technologies, Singapore).

Detection of common respiratory pathogens in HMPV-positive patients

HMPV-positive samples were tested simultaneously using TaqMan Real-time PCR assays for the following 17 respiratory pathogens: respiratory syncytial virus (RSV); parainfluenza virus types 1–4 (PIV1–4); influenza A and B viruses (InfA, InfB); adenovirus (ADV); enterovirus (EV); human coronaviruses (HCoV-229E, HCoV-OC43, HCoV-NL63, HCoV-HKU1); human rhinovirus (HRV); human bocavirus (HBoV); *Mycoplasma pneumoniae* (MP); and *Chlamydophila pneumoniae* (CP); The testing procedure was conducted using kits from Guangzhou HuYanSuo Medical Technology Co., Ltd as previously described.[18]

Statistical analysis

All statistical analyses were performed with SPSS statistical software (version 19.0; SPSS Inc., Chicago, IL, USA). To compare categorical data, χ^2 and Fisher's exact tests were used, as appropriate. All tests were two-tailed, and p<0.05 was considered statistically significant.

For peer review only - http://bmjopen.bmj.com/site/about/guidelines.xhtml

Results

Detection of HMPV from patients with ARI

In total, 5133 pediatric patients, ranging from 1 day to 14 years old were enrolled in this study. The male-to-female ratio was 1.75 (3269:1864) and the median age was 33 months (interquartile range, 9–48 months). Of the 5133 patients, 103 (2.0%) were positive for HMPV. The male-to-female ratio was 1.86:1 (67:36) in HMPV-positive patients and 1.75:1 (3202:1828) in HMPV-negative patients (p=0.771). The median age in months of HMPV-positive patients was 23.5 months (interquartile range, 10–36 months).

Co-detection with common respiratory pathogens in HMPV-positive patients

We also tested HMPV-positive samples for 17 common respiratory pathogens. Of the 103 HMPV-positive patients, 84 (81.6%) patients had only HMPV detection, and 19 (18.4%) patients had HMPV plus other pathogen(s) detected. Nine out of 17 respiratory pathogens (52.9%) were detected and the most common co-detection pathogens were RSV (36.8%, 7/19), HCoV-OC43 (15.8%, 3/19), MP (15.8%, 3/19), and HBoV (15.8%, 3/19) (**Table 1**). The male-to-female ratio was 2:1 (56:28) in patients with only HMPV detected and 1.38:1 (11:8) in patients with HMPV plus other pathogen(s) detected (p=0.469).

Age distribution of HMPV-positive patients

Overall, there was a significant difference in HMPV prevalence between

For peer review only - http://bmjopen.bmj.com/site/about/guidelines.xhtml

patients >5–14 years (0.7%, 5/734) and those ≤5 years (2.2%, 98/4399) (*p*=0.004). The patients were divided into six age groups: 0–3 months, >3–6 months, >6–12 months, >1–2 years, >2–5 years, and >5–14 years. The distribution of HMPV prevalence between these age groups was significantly different (*p*=0.03), and children >1–2 years had the highest prevalence (2.8%, 21/752) (**Figure 1**).

Seasonal distribution of HMPV

Over the 3-year study period, the prevalence of HMPV peaked twice every year (**Figure 2**). Large peaks occurred in March 2014 (8%, 16/200), May 2015 (7.6%, 8/105) and February 2016 (8.7%, 9/103). Small peaks occurred in November 2014 (1.7%, 2/121), September 2015 (2.8%, 2/72) and May 2016 (1.5%, 2/135) (**Figure 2**).

Clinical presentation of HMPV-positive patients

We analyzed the clinical presentation of 101 of the 103 (98.1%) HMPV-positive patients, the other two patients were excluded from this analysis because of incomplete clinical data. The main clinical features of HMPV-positive patients included cough (100.0%, 101/101), abnormal pulmonary breath sound (91.1%, 92/101), fever (88.1%, 89/101), expectoration (77.2%, 78/101), coryza (50.5%, 51/101) and wheezing (46.5%, 47/101). The main diagnosis by chest radiography was bronchopneumonia (55.4%, 56/101), followed by pneumonia (10.9%, 11/101) and bronchiolitis (7.9%, 8/101) (**Table 2**). We also compared the clinical features of patients with only HMPV detected and those of the patients with HMPV plus other

For peer review only - http://bmjopen.bmj.com/site/about/guidelines.xhtml

pathogen(s) detected. Fever and diarrhea were the two symptoms that were identified as statistically different between these two groups; A fever (≥38˚C) was seen more common in patients with only HMPV detected (91.6%,76/83) , compared with 72.2% (13/18) in patients with HMPV plus other pathogen(s) detected (p=0.037). Conversely, diarrhea appeared more often in patients with HMPV plus other pathogen(s) detected (22.2%, 4/18) than in the patients with only HMPV detected (3.6%, 3/83) (p=0.018) (**Table 2**).

For peer review only

For peer review only - http://bmjopen.bmj.com/site/about/guidelines.xhtml

Discussion

Viruses are the most frequent cause of respiratory infections.[20] HMPV has been recognized as an important cause of ARI since its discovery in 2001. A previous study reported HMPV was responsible for approximately 5–10% of hospitalizations in children suffering from ARI,[14] creating a considerable clinical and economic burden worldwide. HMPV detection rates vary according to geographic location, and the incidence of HMPV may show seasonal or annual patterns in the same area. In previous studies,[21-30] HMPV was detected in approximately 1%–17% of ARI cases. In this 3 year study, 103 of 5133 (2.0%) patients were positive for HMPV, which is similar to previous reports of HMPV in patients with ARI in southern China[31] and Japan.[32]

Children under 5 years of age are most susceptible to HMPV infection, and those younger than 2 years of age are at the greatest risk of developing serious conditions.[15] In our study, HMPV-positive cases appeared more frequently in patients ≤5 year-old (2.2%, 98/4399) than in patients >5 year-old (0.7%, 5/734) (p=0.004), which is consistent with many previous studies.[3 30 33 34] Specifically, HMPV prevalence was highest in children >1–2 years (2.8%, 21/752). This may be explained by the immunity against HMPV, which is passed down from mother-to-child, gradually subsiding over time. Moreover, children were more contact with outside world as they grew older. Therefore, children of this age need more attention to prevent HMPV infection. Moreover, our findings suggested no difference in risk between sex, there was no significant difference in the HMPV prevalence between male and female patients (p=0.771), which is consistent with previous studies.[35 36]

For peer review only - http://bmjopen.bmj.com/site/about/guidelines.xhtml

HMPV has a typical seasonal distribution. HMPV activity is largely affected by different local climate factors. The prevalence of HMPV in temperate climates peaks at the end of winter or in early spring.[37 38] In our study, seasonal peaks of HMPV were detected in February 2016, March 2014 and May 2015. This finding indicates that HMPV circulates primarily during the spring and early summer in Guangzhou, this pattern is similar to previous reports from other subtropical areas.[22 39] Furthermore, we also found small peaks in November 2014, September 2015, and May 2016, the reason for this is unknown. It should be noted that the seasonal distribution of HMPV overlaps with RSV in this geographical area.[19] Knowing the epidemiological characteristics of HMPV will help public health authorities and clinicians to improve strategies for controlling HMPV infection.

HMPV co-infection with other respiratory pathogens has been reported in many studies, including RSV,[40] InfA,[41] InfB,[42] PIV,[43] ADV,[43 44] HBoV,[45] HCoV ,[43 46] HRV,[43] EV,[43] MP,[41] and CP.[41] In our study, most HMPV-positive patients only had HMPV (81.6%, 84/103), and there was no significant difference in the HMPV prevalence according to sex between the patients with only HMPV detected and the patients with HMPV plus other pathogen(s) detected (p=0.469). The co-detection rate was 18.4% (19/103), and RSV (36.8%, 7/19) was the most frequently co-detected pathogen, which might be because of the overlapping seasonal distribution of these two pathogens.[47] Co-detection rates of HCoV-OC43, MP, HBoV, ADV, HRV, InfA, PIV2, and PIV3 were over 5%, suggesting a broad range of respiratory pathogens could co-exist with HMPV in ARI pediatric patients.

For peer review only - http://bmjopen.bmj.com/site/about/guidelines.xhtml

HMPV most commonly causes URTI and LRTI in young children. The clinical manifestations of HMPV-positive patients are similar to those of RSV-positive patients, especially in young children.[48-50] In previous studies, the most frequent diagnoses of children hospitalized with HMPV infection were pneumonitis and bronchiolitis.[3 51 52] In some studies, bronchiolitis and recurrent wheezing/pneumonia were the main clinical diagnoses,[7 22 53 54] while in other studies, bronchopneumonia, bronchiolitis, and bronchial asthma exacerbation were the main clinical diagnoses.[34] Our study showed that 55.4% (56/101), 7.9% (8/101) and 10.9% (11/101) of HMPV-positive patients were diagnosed with bronchopneumonia, bronchiolitis, and pneumonia by chest radiography, respectively. Of all the clinical features recorded, cough (100.0%, 101/101), abnormal pulmonary breath sound (91.1%, 92/101), fever (88.1%, 89/101), expectoration (77.2%, 78/101), coryza (50.5%, 51/101), and wheezing (46.5%, 47/101) occurred frequently in HMPV-positive patients, similar to those reported previously.[16 29 52 55]

Most of the HMPV-positive patients had a fever (88.1%, 89/101); it appeared more frequently in patients with only HMPV detected than in the patients with HMPV plus other pathogen(s) detected (*p*=0.037). Diarrhea was not a major symptom in HMPV-positive patients (7.0%, 7/101), but it had a higher prevalence in patients with HMPV plus other pathogen(s) detected than in the patients with only HMPV detected (*p=0.018*), suggesting that diarrhea is probably caused by other pathogens. No significant difference was found in the other clinical features between the patients with only HMPV detected and the patients with HMPV plus other pathogen(s) detected,

For peer review only - http://bmjopen.bmj.com/site/about/guidelines.xhtml

similar to the results of a previous study.[51]

In our study, RSV (36.8%, 7/19) was the most frequently co-detected pathogen in HMPV-positive patients. Whether HMPV/RSV co-infection is more severe than the respective mono-infections remains unclear. A previous study showed that children with HMPV/RSV co-infection were more likely to develop pneumonia; however, disease severity was not increased.[53] Conversely, Semple et al.[56] reported that HMPV/RSV co-infection can cause more severe bronchiolitis in patients. In our study, no significant difference in the diagnosis of pneumonia was observed between the patients with only HMPV detected and the patients with HMPV plus other pathogen(s) detected. It has been confirmed that a history of prematurity, particular age groups, and the presence of chronic diseases increases the risk of severe LRTI among HMPV- and RSV-infected children.[51] Our study suggests that the clinical manifestations of HMPV infection are complex and diverse; our data will be helpful in the diagnosis of HMPV.

The study had some limitations. First, collection of data on symptoms and physical findings in infants and young children requires experienced medical staff, patient's cooperation, and consent from the patient’s guardian. Consequently, it is possible we will have incomplete or even an inaccurate description of patients’ manifestations. Second, because our study mainly focused on HMPV, other common respiratory pathogens, including bacterial pathogens, were not tested and subsequently our study does not give a fully account of respiratory pathogen infection in hospitalized pediatric patients with ARI. Third, the characteristics of HMPV-negative

For peer review only - http://bmjopen.bmj.com/site/about/guidelines.xhtml

patients and outcome data (discharge/death) were not analyzed further, because our study only focused on the epidemiological and clinical features of HMPV positive cases. This might affect our understanding of the clinical features of HMPV-positive patients. Despite these shortcomings, our results provide a valuable insight into the epidemiological and clinical characteristics of HMPV present in patients hospitalized with ARI in Guangzhou, southern China.

For peer review only

For peer review only - http://bmjopen.bmj.com/site/about/guidelines.xhtml

Conclusions

HMPV is an important respiratory pathogen in children with ARI in Guangzhou , China, particularly in children under 5 years old. In future, our data can be used by public health authorities and clinicians to improve the management of HMPV infection in children.

For peer review only

For peer review only - http://bmjopen.bmj.com/site/about/guidelines.xhtml

Acknowledgements

We thank the study volunteers for their generous participation. We thank Yinghua Zhou, Haiping Huang, Jing Zhang, and Jing Ma for technical assistance. We thank Professor Xiaofeng Li and Dr. Zhengshi Lin for paper polishing.

Contributors

RZ, WKL, LZ and DLL designed the study. LZ, DLL, WKL, SYQ, DX, XL and LTT performed pathogen testing. DHC and WPT collected the clinical data. All authors participated in the data analysis. LZ, DLL, RZ and WKL drafted the manuscript. All authors read and approved the final version of this manuscript.

Funding

This study was supported by The State Major Infectious Disease Research Program (RZ) (2017ZX10103011-003), National Natural Science Foundation of China (WKL) (31500143), Guangzhou Science and Technology Program key projects (RZ) (201508020252). The funders had no role in study design, data collection and analysis, decision to publish, or preparation of the manuscript.

Competing interests

None declared.

Ethics approval

For peer review only - http://bmjopen.bmj.com/site/about/guidelines.xhtml

The study was approved by The First Affiliated Hospital of Guangzhou Medical University Ethics Committee. Informed written consent was obtained from parents or legal guardians.

Data sharing statement

No additional data are available.

For peer review only

References

1. van den Hoogen BG, Herfst S, Sprong L, et al. Antigenic and genetic variability of human metapneumoviruses. *Emerg Infect Dis* 2004;10(4):658-66. doi: 10.3201/eid1004.030393

2. van den Hoogen BG, de Jong JC, Groen J, et al. A newly discovered human pneumovirus isolated from young children with respiratory tract disease. *Nat Med* 2001;7(6):719-24. doi: 10.1038/89098

3. Boivin G, Abed Y, Pelletier G, et al. Virological features and clinical manifestations associated with human metapneumovirus: a new paramyxovirus responsible for acute respiratory-tract infections in all age groups. *J Infect Dis* 2002;186(9):1330-4. doi: 10.1086/344319

4. Falsey AR, Walsh EE. Viral pneumonia in older adults. *Clin Infect Dis* 2006;42(4):518-24. doi: 10.1086/499955

5. Kahn JS. Epidemiology of human metapneumovirus. *Clin Microbiol Rev* 2006;19(3):546-57. doi: 10.1128/CMR.00014-06

6. Davis CR, Stockmann C, Pavia AT, et al. Incidence, Morbidity, and Costs of Human Metapneumovirus Infection in Hospitalized Children. *J Pediatric Infect Dis Soc* 2016;5(3):303-11. doi: 10.1093/jpids/piv027

7. Edwards KM, Zhu Y, Griffin MR, et al. Burden of human metapneumovirus infection in young children. *N Engl J Med* 2013;368(7):633-43. doi: 10.1056/NEJMoa1204630

8. Foulongne V, Guyon G, Rodiere M, et al. Human metapneumovirus infection

For peer review only - http://bmjopen.bmj.com/site/about/guidelines.xhtml

in young children hospitalized with respiratory tract disease. *Pediatr Infect Dis J* 2006;25(4):354-9. doi: 10.1097/01.inf.0000207480.55201.f6

9. Heikkinen T, Osterback R, Peltola V, et al. Human metapneumovirus infections in children. *Emerg Infect Dis* 2008;14(1):101-6. doi: 10.3201/eid1401.070251

10. Nicholson KG, McNally T, Silverman M, et al. Rates of hospitalisation for influenza, respiratory syncytial virus and human metapneumovirus among infants and young children. *Vaccine* 2006;24(1):102-8. doi: 10.1016/j.vaccine.2005.02.004

11. Sloots TP, Mackay IM, Bialasiewicz S, et al. Human metapneumovirus, Australia, 2001-2004. *Emerg Infect Dis* 2006;12(8):1263-6. doi: 10.3201/eid1208.051239

12. Williams JV, Edwards KM, Weinberg GA, et al. Population-based incidence of human metapneumovirus infection among hospitalized children. *J Infect Dis* 2010;201(12):1890-8. doi: 10.1086/652782

13. Ebihara T, Endo R, Kikuta H, et al. Seroprevalence of human metapneumovirus in Japan. *J Med Virol* 2003;70(2):281-3. doi: 10.1002/jmv.10391

14. Panda S, Mohakud NK, Pena L, et al. Human metapneumovirus: review of an important respiratory pathogen. *Int J Infect Dis* 2014;25:45-52. doi: 10.1016/j.ijid.2014.03.1394

15. Broor S, Bharaj P, Chahar HS. Human metapneumovirus: a new respiratory

For peer review only

For peer review only - http://bmjopen.bmj.com/site/about/guidelines.xhtml

pathogen. *J Biosci* 2008;33(4):483-93.

16. Jain B, Singh AK, Dangi T, et al. High prevalence of human metapneumovirus subtype B in cases presenting as severe acute respiratory illness: an experience at tertiary care hospital. *Clin Respir J* 2014;8(2):225-33. doi: 10.1111/crj.12064

17. Liu WK, Liu Q, Chen DH, et al. Epidemiology and clinical presentation of the four human parainfluenza virus types. *BMC Infect Dis* 2013;13:28. doi: 10.1186/1471-2334-13-28

18. Liu WK, Chen DH, Liu Q, et al. Detection of human bocavirus from children and adults with acute respiratory tract illness in Guangzhou, southern China. *BMC Infect Dis* 2011;11:345. doi: 10.1186/1471-2334-11-345

19. Liu W, Chen D, Tan W, et al. Epidemiology and Clinical Presentations of Respiratory Syncytial Virus Subgroups A and B Detected with Multiplex Real-Time PCR. *PLoS One* 2016;11(10):e0165108.DOI: 10.1371/journal.pone.08. doi: 10.1371/journal.pone.0165108

20. Williams BG, Gouws E, Boschi-Pinto C, et al. Estimates of world-wide distribution of child deaths from acute respiratory infections. *Lancet Infect Dis* 2002;2(1):25-32.

21. Escobar C, Luchsinger V, de Oliveira DB, et al. Genetic variability of human metapneumovirus isolated from Chilean children, 2003-2004. *J Med Virol* 2009;81(2):340-4. doi: 10.1002/jmv.21399

22. Garcia-Garcia ML, Calvo C, Rey C, et al. Human metapnuemovirus

For peer review only - http://bmjopen.bmj.com/site/about/guidelines.xhtml

infections in hospitalized children and comparison with other respiratory viruses. 2005-2014 prospective study. *PLoS One* 2017;12(3):e0173504.DOI: 10.1371/journal.pone.04. doi: 10.1371/journal.pone.0173504

23. Ljubin-Sternak S, Santak M, Cepin-Bogovic J, et al. Detection of genetic lineages of human metapneumovirus in Croatia during the winter season 2005/2006. *J Med Virol* 2008;80(7):1282-7. doi: 10.1002/jmv.21196

24. McCracken JP, Arvelo W, Ortiz J, et al. Comparative epidemiology of human metapneumovirus- and respiratory syncytial virus-associated hospitalizations in Guatemala. *Influenza Other Respir Viruses* 2014;8(4):414-21. doi: 10.1111/irv.12251

25. Nor'e SS, Sam IC, Mohamad Fakri EF, et al. Phylogenetic analysis of human metapneumovirus among children with acute respiratory infections in Kuala Lumpur, Malaysia. *Trop Biomed* 2014;31(3):562-6.

26. Ordas J, Boga JA, Alvarez-Arguelles M, et al. Role of metapneumovirus in viral respiratory infections in young children. *J Clin Microbiol* 2006;44(8):2739-42. doi: 10.1128/JCM.00164-06

27. Rafiefard F, Yun Z, Orvell C. Epidemiologic characteristics and seasonal distribution of human metapneumovirus infections in five epidemic seasons in Stockholm, Sweden, 2002-2006. *J Med Virol* 2008;80(9):1631-8. doi: 10.1002/jmv.21242

28. Rodriguez PE, Adamo MP, Paglini MG, et al. [Monoinfection of human Metapneumovirus in Cordoba: first clinical and epidemiological research in

For peer review only - http://bmjopen.bmj.com/site/about/guidelines.xhtml

children with respiratory infection in 2011]. *Rev Fac Cien Med Univ Nac Cordoba* 2016;73(3):170-75.

29. Zeng SZ, Xiao NG, Zhong LL, et al. Clinical features of human metapneumovirus genotypes in children with acute lower respiratory tract infection in Changsha, China. *J Med Virol* 2015;87(11):1839-45. doi: 10.1002/jmv.24249

30. Zhu RN, Qian Y, Zhao LQ, et al. Characterization of human metapneumovirus from pediatric patients with acute respiratory infections in a 4-year period in Beijing, China. *Chin Med J (Engl)* 2011;124(11):1623-8.

31. Cai XY, Wang Q, Lin GY, et al. Respiratory virus infections among children in South China. *J Med Virol* 2014;86(7):1249-55. doi: 10.1002/jmv.23931

32. Matsuzaki Y, Itagaki T, Abiko C, et al. Clinical impact of human metapneumovirus genotypes and genotype-specific seroprevalence in Yamagata, Japan. *J Med Virol* 2008;80(6):1084-9. doi: 10.1002/jmv.21194

33. Barbosa Ramirez J, Pulido Dominguez P, Rey Benito G, et al. Human respiratory syncytial virus and metapneumovirus in patients with acute respiratory infection in Colombia, 2000 - 2011. *Rev Panam Salud Publica* 2014;36(2):101-9.

34. Zhang C, Du LN, Zhang ZY, et al. Detection and genetic diversity of human metapneumovirus in hospitalized children with acute respiratory infections in Southwest China. *J Clin Microbiol* 2012;50(8):2714-9. doi: 10.1128/JCM.00809-12

For peer review only - http://bmjopen.bmj.com/site/about/guidelines.xhtml

35. McAdam AJ, Hasenbein ME, Feldman HA, et al. Human metapneumovirus in children tested at a tertiary-care hospital. *J Infect Dis* 2004;190(1):20-6. doi: 10.1086/421120

36. Boivin G, De Serres G, Cote S, et al. Human metapneumovirus infections in hospitalized children. *Emerg Infect Dis* 2003;9(6):634-40. doi: 10.3201/eid0906.030017

37. Maggi F, Pifferi M, Vatteroni M, et al. Human metapneumovirus associated with respiratory tract infections in a 3-year study of nasal swabs from infants in Italy. *J Clin Microbiol* 2003;41(7):2987-91.

38. van den Hoogen BG, van Doornum GJ, Fockens JC, et al. Prevalence and clinical symptoms of human metapneumovirus infection in hospitalized patients. *J Infect Dis* 2003;188(10):1571-7. doi: 10.1086/379200

39. Peiris JS, Tang WH, Chan KH, et al. Children with respiratory disease associated with metapneumovirus in Hong Kong. *Emerg Infect Dis* 2003;9(6):628-33. doi: 10.3201/eid0906.030009

40. Greensill J, McNamara PS, Dove W, et al. Human metapneumovirus in severe respiratory syncytial virus bronchiolitis. *Emerg Infect Dis* 2003;9(3):372-5. doi: 10.3201/eid0903.020289

41. Lin PY, Lin TY, Huang YC, et al. Human metapneumovirus and community-acquired pneumonia in children. *Chang Gung Med J* 2005;28(10):683-8.

42. Ghattas C, Mossad SB. Fatal human metapneumovirus and influenza B virus

For peer review only - http://bmjopen.bmj.com/site/about/guidelines.xhtml

coinfection in an allogeneic hematopoietic stem cell transplant recipient. *Transpl Infect Dis* 2012;14(5):E41-3.DOI: 10.1111/j.399-3062.2012.00766.x. doi: 10.1111/j.1399-3062.2012.00766.x

43. Fathima S, Lee BE, May-Hadford J, et al. Use of an innovative web-based laboratory surveillance platform to analyze mixed infections between human metapneumovirus (hMPV) and other respiratory viruses circulating in Alberta (AB), Canada (2009-2012). *Viruses* 2012;4(11):2754-65. doi: 10.3390/v4112754

44. Kaida A, Kubo H, Goto K, et al. Co-infection of human metapneumovirus with adenovirus or respiratory syncytial virus among children in Japan. *Microbiol Immunol* 2007;51(7):679-83.

45. Pilger DA, Cantarelli VV, Amantea SL, et al. Detection of human bocavirus and human metapneumovirus by real-time PCR from patients with respiratory symptoms in Southern Brazil. *Mem Inst Oswaldo Cruz* 2011;106(1):56-60.

46. Esposito S, Daleno C, Prunotto G, et al. Impact of viral infections in children with community-acquired pneumonia: results of a study of 17 respiratory viruses. *Influenza Other Respir Viruses* 2013;7(1):18-26. doi: 10.1111/j.1750-2659.2012.00340.x

47. Kahn JS. Newly discovered respiratory viruses: significance and implications. *Curr Opin Pharmacol* 2007;7(5):478-83. doi: 10.1016/j.coph.2007.07.004

48. Wolf DG, Greenberg D, Kalkstein D, et al. Comparison of human metapneumovirus, respiratory syncytial virus and influenza A virus lower

For peer review only - http://bmjopen.bmj.com/site/about/guidelines.xhtml

respiratory tract infections in hospitalized young children. *Pediatr Infect Dis J* 2006;25(4):320-4. doi: 10.1097/01.inf.0000207395.80657.cf

49. Wilkesmann A, Schildgen O, Eis-Hubinger AM, et al. Human metapneumovirus infections cause similar symptoms and clinical severity as respiratory syncytial virus infections. *Eur J Pediatr* 2006;165(7):467-75. doi: 10.1007/s00431-006-0105-4

50. Adams O, Weis J, Jasinska K, et al. Comparison of human metapneumovirus, respiratory syncytial virus and Rhinovirus respiratory tract infections in young children admitted to hospital. *J Med Virol* 2015;87(2):275-80. doi: 10.1002/jmv.24025

51. Moe N, Krokstad S, Stenseng IH, et al. Comparing Human Metapneumovirus and Respiratory Syncytial Virus: Viral Co-Detections, Genotypes and Risk Factors for Severe Disease. *PLoS One* 2017;12(1):e0170200.DOI: 10.1371/journal.pone.00. doi: 10.1371/journal.pone.0170200

52. Yan XL, Li YN, Tang YJ, et al. Clinical characteristics and viral load of respiratory syncytial virus and human metapneumovirus in children hospitaled for acute lower respiratory tract infection. *J Med Virol* 2017;89(4):589-97. doi: 10.1002/jmv.24687

53. Caracciolo S, Minini C, Colombrita D, et al. Human metapneumovirus infection in young children hospitalized with acute respiratory tract disease: virologic and clinical features. *Pediatr Infect Dis J* 2008;27(5):406-12. doi: 10.1097/INF.0b013e318162a164

For peer review only

For peer review only - http://bmjopen.bmj.com/site/about/guidelines.xhtml

54. Williams JV, Harris PA, Tollefson SJ, et al. Human metapneumovirus and lower respiratory tract disease in otherwise healthy infants and children. *N Engl J Med* 2004;350(5):443-50. doi: 10.1056/NEJMoa025472

55. Schuster JE, Khuri-Bulos N, Faouri S, et al. Human Metapneumovirus Infection in Jordanian Children: Epidemiology and Risk Factors for Severe Disease. *Pediatr Infect Dis J* 2015;34(12):1335-41. doi: 10.1097/INF.0000000000000892

56. Semple MG, Cowell A, Dove W, et al. Dual infection of infants by human metapneumovirus and human respiratory syncytial virus is strongly associated with severe bronchiolitis. *J Infect Dis* 2005;191(3):382-6. doi: 10.1086/426457

For peer review only

For peer review only - http://bmjopen.bmj.com/site/about/guidelines.xhtml

Figure legends

Figure 1 Age distributions of patients with HMPV.

[a]Date were presented as HMPV positive rate (number of HMPV-positive patients/number of patients in each age group); m: month(s); y: year(s).

Figure 2 Seasonal distribution of HMPV infection in pediatric patients hospitalized with acute respiratory infection from July 2013 to June 2016 in Guangzhou.

For peer review only

For peer review only - http://bmjopen.bmj.com/site/about/guidelines.xhtml

Table 1. Distribution of co-pathogens in 19 HMPV-positive patients

Co-pathogens[a]	Patient No.
RSV/HMPV	4
HCoV-OC43/HMPV	3
ADV/HMPV	2
HRV/HMPV	2
MP/HMPV	1
PIV2/HMPV	1
PIV3/HMPV	1
RSV/MP/HMPV	1
RSV/InfA/HMPV	1
RSV/HBoV/HMPV	1
MP/HBoV/HMPV	1
InfA/HBoV/HMPV	1

[a]HMPV-positive patients were tested for 17 common respiratory pathogens.

RSV: respiratory syncytial virus; PIV: parainfluenza virus; InfA: influenza A virus; ADV: adenovirus; HCoV: human coronaviruses; HRV: human rhinovirus; HBoV: human bocavirus; MP: *Mycoplasma pneumoniae.*

Influenza B viruses, parainfluenza virus types 1 and 4, enterovirus, human coronaviruses-HKU1, human coronaviruses-229E, human coronaviruses-NL63, and *Chlamydophila pneumoniae* were not detected.

For peer review only - http://bmjopen.bmj.com/site/about/guidelines.xhtml

527 **Table 2. Clinical presentations of HMPV-positive patients**

Diagnosis/Symptom	Total HMPV (n=101)	Patients with only HMPV and HMPV plus other pathogen(s) detected.		
		Single HMPV (n = 83)	Co-pathogens (n= 18)	p value[b]
**Upper respiratory tract infection**				
Nasal obstruction	40(39.6)	34(41.0)	6(33.3)	0.605
Coryza	51(50.5)	44(53.0)	7(38.9)	0.309
Sneeze	1(1.0)	1(1.2)	0(0)	–[c]
Cough	101(100)	83(100)	18(100)	–[d]
Expectoration	78(77.2)	66(79.5)	12(66.7)	0.351
Hoarseness	1(1.0)	1(1.2)	0(0)	–[c]
**Lower respiratory tract infection**				
Abnormal pulmonary breath sound [a]	92(91.1)	75(90.4)	17(94.4)	0.582
Increasing lung markings	6(6.0)	4(4.8)	2(11.1)	0.29
wheezing	47(46.5)	40(48.2)	7(38.9)	0.473
anhelation	24(23.8)	21(25.3)	3(16.7)	0.551
Bronchiolitis	8(7.9)	7(8.4)	1(5.6)	0.682
Pneumonia	11(10.9)	10(12.1)	1(5.6)	0.684
bronchopneumonia	56(55.4)	46(55.4)	10(55.6)	0.992
**Systemic influenza-like symptoms**				
Fever (≥38˚C)	89(88.1)	76(91.6)	13(72.2)	**0.037**
Chill	7(6.9)	6(7.2)	1(5.6)	0.8
Debilitation	11(10.9)	11(13.3)	0(0)	–[c]
**Gastrointestinal illness**				
Vomiting	21(20.8)	16(19.3)	5(27.8)	0.522
Poor appetite	19(18.8)	17(20.5)	2(11.1)	0.513
Diarrhea	7(7.0)	3(3.6)	4(22.2)	**0.018**

528 Data are presented as No. (%) of each group.

529 Percentages sum to >100% because some patients had >1 clinical presentations.

530 [a]Including phlegmatic, wheezing, bubbling, and moist rales.

531 [b]Two-tailed χ^2 test comparing the distribution of each illness or diagnosis between

532 patients with only HMPV and HMPV plus other pathogen(s) detected.

533 [c]Not tested because the number of positive samples obtained was too small.

534 [d]Not tested because the prevalence was 100%.

For peer review only - http://bmjopen.bmj.com/site/about/guidelines.xhtml

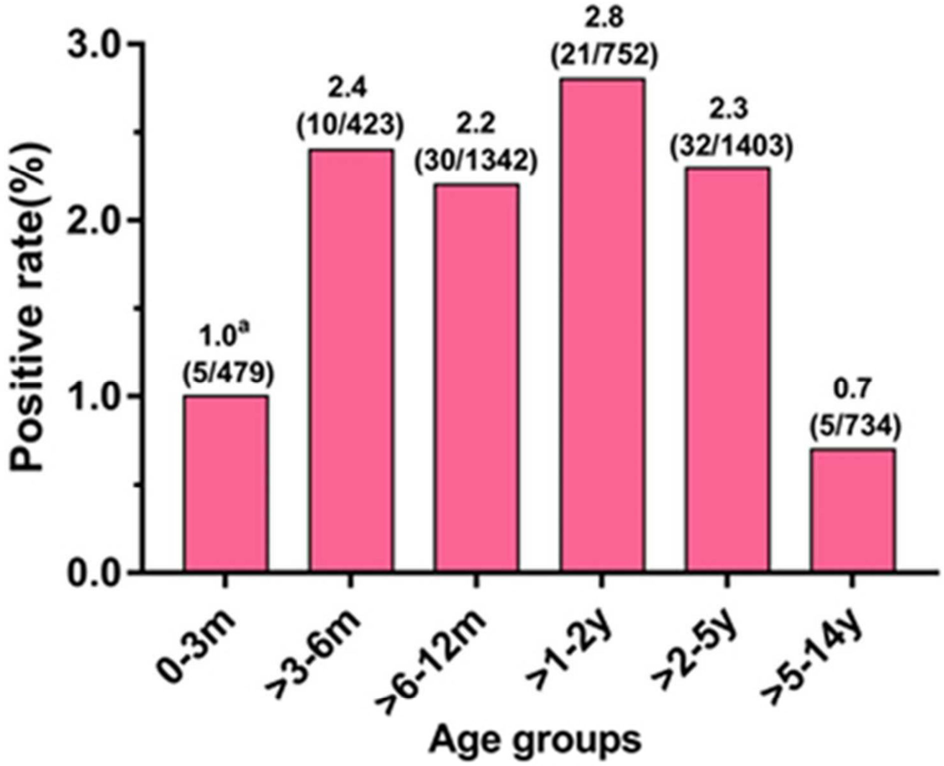

Figure 1 Age distributions of patients with HMPV.
aDate were presented as HMPV positive rate (number of HMPV-positive patients/number of patients in each age group); m: month(s); y: year(s).

42x35mm (300 x 300 DPI)

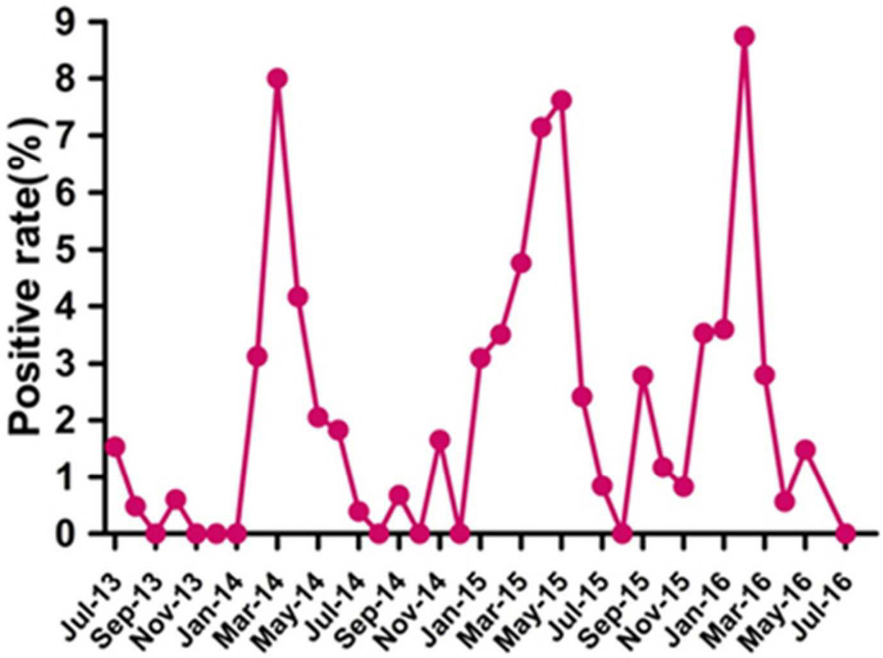

Figure 2 Seasonal distribution of HMPV infection in pediatric patients hospitalized with acute respiratory infection from July 2013 to June 2016 in Guangzhou.

39x31mm (300 x 300 DPI)

STROBE 2007 (v4)Statement—Checklist of items that should be included in reports of *cross-sectional studies*

Section/Topic	Item #	Recommendation	Reported on page #
**Title and abstract**	1	(*a*) Indicate the study's design with a commonly used term in the title or the abstract	1
		(*b*) Provide in the abstract an informative and balanced summary of what was done and what was found	2-3
**Introduction**			
Background/rationale	2	Explain the scientific background and rationale for the investigation being reported	4
Objectives	3	State specific objectives, including any prespecified hypotheses	4-5
**Methods**			
Study design	4	Present key elements of study design early in the paper	6
Setting	5	Describe the setting, locations, and relevant dates, including periods of recruitment, exposure, follow-up, and data collection	6
Participants	6	(*a*) Give the eligibility criteria, and the sources and methods of selection of participants	6
Variables	7	Clearly define all outcomes, exposures, predictors, potential confounders, and effect modifiers. Give diagnostic criteria, if applicable	6-7
Data sources/ measurement	8*	For each variable of interest, give sources of data and details of methods of assessment (measurement). Describe comparability of assessment methods if there is more than one group	7
Bias	9	Describe any efforts to address potential sources of bias	*6*
Study size	10	Explain how the study size was arrived at	6
Quantitative variables	11	Explain how quantitative variables were handled in the analyses. If applicable, describe which groupings were chosen and why	7-8
Statistical methods	12	(*a*) Describe all statistical methods, including those used to control for confounding	8
		(*b*) Describe any methods used to examine subgroups and interactions	8
		(*c*) Explain how missing data were addressed	7
		(*d*) If applicable, describe analytical methods taking account of sampling strategy	8
		(*e*) Describe any sensitivity analyses	
**Results**			

For peer review only

Participants	13*	(a) Report numbers of individuals at each stage of study—eg numbers potentially eligible, examined for eligibility, confirmed eligible, included in the study, completing follow-up, and analysed	9
		(b) Give reasons for non-participation at each stage	10
		(c) Consider use of a flow diagram	
Descriptive data	14*	(a) Give characteristics of study participants (eg demographic, clinical, social) and information on exposures and potential confounders	9-11
		(b) Indicate number of participants with missing data for each variable of interest	9
Outcome data	15*	Report numbers of outcome events or summary measures	9-11
Main results	16	(*a*) Give unadjusted estimates and, if applicable, confounder-adjusted estimates and their precision (eg, 95% confidence interval). Make clear which confounders were adjusted for and why they were included	9-11
		(*b*) Report category boundaries when continuous variables were categorized	9-10
		(*c*) If relevant, consider translating estimates of relative risk into absolute risk for a meaningful time period	
Other analyses	17	Report other analyses done—eg analyses of subgroups and interactions, and sensitivity analyses	
**Discussion**			
Key results	18	Summarise key results with reference to study objectives	12-15
Limitations	19	Discuss limitations of the study, taking into account sources of potential bias or imprecision. Discuss both direction and magnitude of any potential bias	15-16
Interpretation	20	Give a cautious overall interpretation of results considering objectives, limitations, multiplicity of analyses, results from similar studies, and other relevant evidence	12-16
Generalisability	21	Discuss the generalisability (external validity) of the study results	12-16
**Other information**			
Funding	22	Give the source of funding and the role of the funders for the present study and, if applicable, for the original study on which the present article is based	18

*Give information separately for cases and controls in case-control studies and, if applicable, for exposed and unexposed groups in cohort and cross-sectional studies.

Note: An Explanation and Elaboration article discusses each checklist item and gives methodological background and published examples of transparent reporting. The STROBE checklist is best used in conjunction with this article (freely available on the Web sites of PLoS Medicine at http://www.plosmedicine.org/, Annals of Internal Medicine at http://www.annals.org/, and Epidemiology at http://www.epidem.com/). Information on the STROBE Initiative is available at www.strobe-statement.org.

For peer review only

Epidemiology and clinical features of human metapneumovirus in hospitalized pediatric patients with acute respiratory illness: a cross-sectional study in southern China, from 2013 to 2016

Journal:	*BMJ Open*
Manuscript ID	bmjopen-2017-019308.R3
Article Type:	Research
Date Submitted by the Author:	27-Dec-2017

Complete List of Authors:	Zhang, Ling; State Key Laboratory of Respiratory Diseases, The First Affiliated Hospital of Guangzhou Medical University, Guangzhou Medical University<br>Liu, Wenkuan; State Key Laboratory of Respiratory Diseases, The First Affiliated Hospital of Guangzhou Medical University, Guangzhou Medical University<br>Liu, Donglan; State Key Laboratory of Respiratory Diseases, The First Affiliated Hospital of Guangzhou Medical University, Guangzhou Medical University<br>Chen, Dehui; Department of Pediatrics, The First Affiliated Hospital of Guangzhou Medical University, Guangzhou Medical University<br>Tan, Weiping ; Sun Yat-Sen Memorial Hospital, Sun Yat-Sen University<br>Qiu, Shuyan; State Key Laboratory of Respiratory Diseases, The First Affiliated Hospital of Guangzhou Medical University, Guangzhou Medical University<br>Xu, Duo; State Key Laboratory of Respiratory Diseases, The First Affiliated Hospital of Guangzhou Medical University, Guangzhou Medical University<br>Li, Xiao; State Key Laboratory of Respiratory Diseases, The First Affiliated Hospital of Guangzhou Medical University, Guangzhou Medical University<br>Liu, Tiantian; State Key Laboratory of Respiratory Diseases, The First Affiliated Hospital of Guangzhou Medical University, Guangzhou Medical University<br>Zhou, Rong; State Key Laboratory of Respiratory Diseases, The First Affiliated Hospital of Guangzhou Medical University, Guangzhou Medical University

**Primary Subject Heading**:	Infectious diseases
Secondary Subject Heading:	Epidemiology, Paediatrics, Infectious diseases
Keywords:	Respiratory infections < THORACIC MEDICINE, Epidemiology < INFECTIOUS DISEASES, VIROLOGY, Diagnostic microbiology < INFECTIOUS DISEASES

SCHOLARONE™
Manuscripts

For peer review only

Epidemiology and clinical features of human metapneumovirus in hospitalized pediatric patients with acute respiratory illness: a cross-sectional study in southern China, from 2013 to 2016

Ling Zhang[1†], Wenkuan Liu[1†], Donglan Liu[1†], Dehui Chen[2], Weiping Tan[3], Shuyan Qiu[1], Duo Xu[1], Xiao Li[1], Tiantian Liu[1], Rong Zhou[1]*

[1]State Key Laboratory of Respiratory Diseases, The First Affiliated Hospital of Guangzhou Medical University, Guangzhou Medical University, Guangzhou, People's Republic of China.

[2]Department of Pediatrics, The First Affiliated Hospital of Guangzhou Medical University, Guangzhou Medical University, Guangzhou, People's Republic of China.

[3]Sun Yat-Sen Memorial Hospital, Sun Yat-Sen University, Guangzhou, People's Republic of China.

[†]Ling Zhang, Wenkuan Liu and Donglan Liu contributed equally to this work.

*Correspondence to Rong Zhou, e-mail: zhourong@gird.cn

For peer review only - http://bmjopen.bmj.com/site/about/guidelines.xhtml

Abstract

Objectives: Human metapneumovirus (HMPV) is one of the most important respiratory viral pathogens affecting infants and children worldwide. Our study describes the epidemiological and clinical characteristics of HMPV present in patients hospitalized with acute respiratory illness (ARI) in Guangzhou, southern China.

Study design: A cross-sectional study.

Setting: Two tertiary hospitals in Guangzhou.

Participants and methods: Throat swabs were collected over a 3-year period from 5133 pediatric patients (≤14 years) hospitalized with ARI. HMPV-positive patients' clinical presentations (101/103) were recorded for further analysis.

Results: Of the 5133 patients included in the study, 103 (2.0%) were positive for HMPV. HMPV was more prevalent in children ≤5 years (2.2%, 98/4399) compared with older children (>5–14 years) (0.7%, 5/734) (p=0.004). Two seasonal HMPV peaks were observed each year and mainly occurred in spring and early summer. Overall, 18.4% (19/103) of HMPV-positive patients were co-detected with other pathogens, most frequently respiratory syncytial virus (36.8%, 7/19). HMPV-positive patients presented with a wide spectrum of clinical features, including cough (100.0%, 101/101), abnormal pulmonary breath sound (91.1%, 92/101), fever (88.1%, 89/101), expectoration (77.2%, 78/101), coryza (50.5%, 51/101) and wheezing (46.5%, 47/101). The main diagnosis of HMPV-positive patients was bronchopneumonia (66.7%, 56/84). Fever (≥38˚C) (91.6%, 76/83) was detected more often in patients with only HMPV detected than in patients with HMPV plus other pathogen(s) detected

For peer review only - http://bmjopen.bmj.com/site/about/guidelines.xhtml

(72.2%, 13/18) (p=0.037), whereas diarrhea was more common in patients with HMPV plus other pathogen(s) detected (22.2%, 4/18), compared with patients with HMPV only (3.6%, 3/83) (p=0.018).

Conclusions: HMPV is an important respiratory pathogen in children with ARI in Guangzhou, particularly in children ≤5 years old. HMPV has a seasonal variation. Bronchopneumonia is a major diagnosis in HMPV-positive patients.

For peer review only

Strengths and limitations of this study

- 5133 patients hospitalized with acute respiratory illness at two large municipal tertiary hospitals (hospital beds ＞1000) were enrolled on the study over 3 years.
- Patients aged from 1 day to 14 years old were tested for HMPV using Taqman real-time PCR.
- Clinical characteristics of patients with HMPV-positive were recorded.
- There was incomplete HMPV co-pathogen detection, because bacterial pathogens were not detected for HMPV-positive patients.
- The clinical characteristics of HMPV-negative patients and outcome data (discharge/death) for the HMPV-positive patients were not collected for further analysis, limiting our understanding of this pathogen.

Introduction

Human metapneumovirus (HMPV) is a nonsegmented, negative-sense single-stranded RNA virus, which belongs to the *Paramyxoviridae* family.[1] HMPV was

For peer review only - http://bmjopen.bmj.com/site/about/guidelines.xhtml

first discovered in 2001 in the Netherlands, after being isolated from a pediatric patient with acute respiratory illness (ARI).[2] Since then, HMPV has been associated with acute respiratory disease in individuals of all ages worldwide. Children, elderly and immunocompromised adults are most at risk of contracting the virus.[3-5] Children younger than 5 years of age seem to be particularly susceptible to HMPV. Previous studies,[6-12] which were mainly conducted in developed countries, have revealed that approximately 2.5%–11.3% of respiratory samples were positive for HMPV in children ≤5 years, and 90% of individuals were seropositive for HMPV by 5 years of age.[13]

HMPV causes a variety of clinical symptoms ranging from a mild upper respiratory tract infection (URTI) to life-threatening lower respiratory tract infection (LRTI).[3 14-16] However, to date, there is no effective vaccine or specific medication either for prevention or treatment of HMPV infection. Consequently, it is imperative to conduct more studies, especially in low and middle-income countries, to understand this pathogen in different areas and populations.

In this study, we investigated the epidemiological and clinical features of HMPV in pediatric patients, from July 2013 to June 2016. The findings of this study will help understand the distribution of HMPV in a subtropical region. Our results also provide a valuable insight into the clinical features of HMPV, which will improve early clinical diagnosis.

For peer review only - http://bmjopen.bmj.com/site/about/guidelines.xhtml

Methods

Study design and respiratory sample collection

We conducted a cross-sectional study at two tertiary hospitals in Guangzhou, southern China. Throat swab samples (n=5133) from pediatric patients (≤14 years) hospitalized with ARI, were collected at two hospitals between July 2013 and June 2016. ARI was defined as an illness that presented with at least two of the following clinical presentations: cough, nasal obstruction, coryza, sneeze, dyspnoea during the previous week. Patients, who were diagnosed with pneumonia by chest radiography during the previous week, were also included in the study, even if they did not show the clinical features described above. Some patients, who had been cured and discharged some time ago and readmitted because of new episodes of ARI, if met the recruitment criteria, were included in the study as new cases, otherwise excluded. Chest radiography was performed based on the clinical situation of the patients. The samples were collected according to established clinical protocols.[17] The samples were refrigerated at 2–8°C in viral transport medium, transported on ice to the State Key Laboratory of Respiratory Diseases, and analyzed immediately or stored at -80°C before analysis as previously described.[18]

The patients' clinical presentations or diagnoses were recorded from patients' medical records by attending physicians, using designed presentation cards and were categorized retrospectively into the following four groups: URTI, LRTI, systemic influenza-like symptoms, and gastrointestinal illness. Patients with nasal obstruction, coryza, sneezing, coughing, expectoration, or hoarseness were categorized as

For peer review only - http://bmjopen.bmj.com/site/about/guidelines.xhtml

having URTI. Patients with bronchiolitis, pneumonia, bronchopneumonia, increased lung markings, dyspnea, or an abnormal pulmonary breath sound were categorized as having LRTI. Patients with a high fever (≥38°C), chills, or debilitation were categorized as having systemic influenza-like symptoms. Patients with vomiting, poor appetite, or diarrhea were categorized as having gastrointestinal illness. Some patients were assigned to several clinical presentation groups. Patients with incomplete clinical data were excluded from this analysis. Increased lung markings, bronchopneumonia, pneumonia, and bronchiolitis were diagnosed by chest radiography. Abnormal pulmonary breath sounds included phlegmatic, wheezy, bubbling, and moist rales. Other clinical symptoms were identified by a general medical examination and clinical descriptions, as previously reported.[19]

Real-time PCR for HMPV detection

RNA was extracted from the throat swab samples with the QIAamp Viral RNA Mini Kit (Qiagen, Shanghai, China), according to the manufacturer's protocols. HMPV was identified by using TaqMan Real-time PCR assays, as previously reported,[18] using kits from Guangzhou HuYanSuo Medical Technology Co., Ltd according to the manufacturer's protocols. In brief, 50 μl RNA were extracted from a 200 μl sample, and real-time PCR was conducted using 25 μl reaction mix, containing M-MLV, Taq polymerase and 5 μl extracted RNA. Cycling conditions included an initial reverse transcription at 55°C for 10 min, incubation at 94°C for 2 min, followed by 40 cycles of 94°C for 10 sec and 55°C for 35 sec (ABI-7500 real-time PCR instrument, Life

For peer review only - http://bmjopen.bmj.com/site/about/guidelines.xhtml

Technologies, Singapore).

Detection of common respiratory pathogens in HMPV-positive patients

HMPV-positive samples were tested simultaneously using TaqMan Real-time PCR assays for the following 17 respiratory pathogens: respiratory syncytial virus (RSV); parainfluenza virus types 1–4 (PIV1–4); influenza A and B viruses (InfA, InfB); adenovirus (ADV); enterovirus (EV); human coronaviruses (HCoV-229E, HCoV-OC43, HCoV-NL63, HCoV-HKU1); human rhinovirus (HRV); human bocavirus (HBoV); *Mycoplasma pneumoniae* (MP); and *Chlamydophila pneumoniae* (CP); The testing procedure was conducted using kits from Guangzhou HuYanSuo Medical Technology Co., Ltd as previously described.[18]

Statistical analysis

All statistical analyses were performed with SPSS statistical software (version 19.0; SPSS Inc., Chicago, IL, USA). To compare categorical data, χ^2 and Fisher's exact tests were used, as appropriate. All tests were two-tailed, and $p<0.05$ was considered statistically significant.

For peer review only - http://bmjopen.bmj.com/site/about/guidelines.xhtml

Results

Detection of HMPV from patients with ARI

In total, 5133 pediatric patients, ranging from 1 day to 14 years old were enrolled in this study. The male-to-female ratio was 1.75 (3269:1864) and the median age was 33 months (interquartile range, 9–48 months). Of the 5133 patients, 103 (2.0%) were positive for HMPV. The male-to-female ratio was 1.86:1 (67:36) in HMPV-positive patients and 1.75:1 (3202:1828) in HMPV-negative patients (*p*=0.771). The median age in months of HMPV-positive patients was 23.5 months (interquartile range, 10–36 months).

Co-detection with common respiratory pathogens in HMPV-positive patients

We also tested HMPV-positive samples for 17 common respiratory pathogens. Of the 103 HMPV-positive patients, 84 (81.6%) patients had only HMPV detection, and 19 (18.4%) patients had HMPV plus other pathogen(s) detected. Nine out of 17 respiratory pathogens (52.9%) were detected and the most common co-detection pathogens were RSV (36.8%, 7/19), HCoV-OC43 (15.8%, 3/19), MP (15.8%, 3/19), and HBoV (15.8%, 3/19) (**Table 1**). The male-to-female ratio was 2:1 (56:28) in patients with only HMPV detected and 1.38:1 (11:8) in patients with HMPV plus other pathogen(s) detected (p=0.469).

Age distribution of HMPV-positive patients

Overall, there was a significant difference in HMPV prevalence between

For peer review only - http://bmjopen.bmj.com/site/about/guidelines.xhtml

patients >5–14 years (0.7%, 5/734) and those ≤5 years (2.2%, 98/4399) (p=0.004). The patients were divided into six age groups: 0–3 months, >3–6 months, >6–12 months, >1–2 years, >2–5 years, and >5–14 years. The distribution of HMPV prevalence between these age groups was significantly different (p=0.03), and children >1–2 years had the highest prevalence (2.8%, 21/752) (**Figure 1**).

Seasonal distribution of HMPV

Over the 3-year study period, the prevalence of HMPV peaked twice every year (**Figure 2**). Large peaks occurred in March 2014 (8%, 16/200), May 2015 (7.6%, 8/105) and February 2016 (8.7%, 9/103). Small peaks occurred in November 2014 (1.7%, 2/121), September 2015 (2.8%, 2/72) and May 2016 (1.5%, 2/135) (**Figure 2**).

Clinical presentation of HMPV-positive patients

We analyzed the clinical presentation of 101 of the 103 (98.1%) HMPV-positive patients, the other two patients were excluded from this analysis because of incomplete clinical data. The main clinical features of HMPV-positive patients included cough (100.0%, 101/101), abnormal pulmonary breath sound (91.1%, 92/101), fever (88.1%, 89/101), expectoration (77.2%, 78/101), coryza (50.5%, 51/101) and wheezing (46.5%, 47/101). Of 84 with a chest radiography, 56 (66.7%) were diagnosed with bronchopneumonia, 11 (13.1%) were pneumonia and 8 (9.5%) were bronchiolitis (**Table 2**). We also compared the clinical features of patients with only HMPV detected and those of the patients with HMPV plus other pathogen(s) detected.

For peer review only - http://bmjopen.bmj.com/site/about/guidelines.xhtml

Fever and diarrhea were the two symptoms that were identified as statistically different between these two groups; A fever (≥38˚C) was seen more common in patients with only HMPV detected (91.6%,76/83) , compared with 72.2% (13/18) in patients with HMPV plus other pathogen(s) detected (p=0.037). Conversely, diarrhea appeared more often in patients with HMPV plus other pathogen(s) detected (22.2%, 4/18) than in the patients with only HMPV detected (3.6%, 3/83) (p=0.018) (**Table 2**).

For peer review only

For peer review only - http://bmjopen.bmj.com/site/about/guidelines.xhtml

Discussion

Viruses are the most frequent cause of respiratory infections.[20] HMPV has been recognized as an important cause of ARI since its discovery in 2001. A previous study reported HMPV was responsible for approximately 5–10% of hospitalizations in children suffering from ARI,[14] creating a considerable clinical and economic burden worldwide. HMPV detection rates vary according to geographic location, and the incidence of HMPV may show seasonal or annual patterns in the same area. In previous studies,[21-30] HMPV was detected in approximately 1%–17% of ARI cases. In this 3 year study, 103 of 5133 (2.0%) patients were positive for HMPV, which is similar to previous reports of HMPV in patients with ARI in southern China[31] and Japan.[32]

Children under 5 years of age are most susceptible to HMPV infection, and those younger than 2 years of age are at the greatest risk of developing serious conditions.[15] In our study, HMPV-positive cases appeared more frequently in patients ≤5 year-old (2.2%, 98/4399) than in patients >5 year-old (0.7%, 5/734) (p=0.004), which is consistent with many previous studies.[3 30 33 34] Specifically, HMPV prevalence was highest in children >1–2 years (2.8%, 21/752). This may be explained by the immunity against HMPV, which is passed down from mother-to-child, gradually subsiding over time. Moreover, children were more contact with outside world as they grew older. Therefore, children of this age need more attention to prevent HMPV infection. Moreover, our findings suggested no difference in risk between sex, there was no significant difference in the HMPV prevalence between male and female patients (p=0.771), which is consistent with previous studies.[35 36]

For peer review only - http://bmjopen.bmj.com/site/about/guidelines.xhtml

HMPV has a typical seasonal distribution. HMPV activity is largely affected by different local climate factors. The prevalence of HMPV in temperate climates peaks at the end of winter or in early spring.[37 38] In our study, seasonal peaks of HMPV were detected in February 2016, March 2014 and May 2015. This finding indicates that HMPV circulates primarily during the spring and early summer in Guangzhou, this pattern is similar to previous reports from other subtropical areas.[22 39] Furthermore, we also found small peaks in November 2014, September 2015, and May 2016, the reason for this is unknown. It should be noted that the seasonal distribution of HMPV overlaps with RSV in this geographical area.[19] Knowing the epidemiological characteristics of HMPV will help public health authorities and clinicians to improve strategies for controlling HMPV infection.

HMPV co-infection with other respiratory pathogens has been reported in many studies, including RSV,[40] InfA,[41] InfB,[42] PIV,[43] ADV,[43 44] HBoV,[45] HCoV ,[43 46] HRV,[43] EV,[43] MP,[41] and CP.[41] In our study, most HMPV-positive patients only had HMPV (81.6%, 84/103), and there was no significant difference in the HMPV prevalence according to sex between the patients with only HMPV detected and the patients with HMPV plus other pathogen(s) detected (p=0.469). The co-detection rate was 18.4% (19/103), and RSV (36.8%, 7/19) was the most frequently co-detected pathogen, which might be because of the overlapping seasonal distribution of these two pathogens.[47] Co-detection rates of HCoV-OC43, MP, HBoV, ADV, HRV, InfA, PIV2, and PIV3 were over 5%, suggesting a broad range of respiratory pathogens could co-exist with HMPV in ARI pediatric patients.

For peer review only - http://bmjopen.bmj.com/site/about/guidelines.xhtml

HMPV most commonly causes URTI and LRTI in young children. The clinical manifestations of HMPV-positive patients are similar to those of RSV-positive patients, especially in young children.[48-50] In previous studies, the most frequent diagnoses of children hospitalized with HMPV infection were pneumonitis and bronchiolitis.[3 51 52] In some studies, bronchiolitis and recurrent wheezing/pneumonia were the main clinical diagnoses,[7 22 53 54] while in other studies, bronchopneumonia, bronchiolitis, and bronchial asthma exacerbation were the main clinical diagnoses.[34] Our study showed that 66.7% (56/84), 9.5% (8/84) and 13.1% (11/84) of HMPV-positive patients were diagnosed with bronchopneumonia, bronchiolitis, and pneumonia by chest radiography, respectively. Of all the clinical features recorded, cough (100.0%, 101/101), abnormal pulmonary breath sound (91.1%, 92/101), fever (88.1%, 89/101), expectoration (77.2%, 78/101), coryza (50.5%, 51/101), and wheezing (46.5%, 47/101) occurred frequently in HMPV-positive patients, similar to those reported previously.[16 29 52 55]

Most of the HMPV-positive patients had a fever (88.1%, 89/101); it appeared more frequently in patients with only HMPV detected than in the patients with HMPV plus other pathogen(s) detected (p=0.037). Diarrhea was not a major symptom in HMPV-positive patients (7.0%, 7/101), but it had a higher prevalence in patients with HMPV plus other pathogen(s) detected than in the patients with only HMPV detected ($p=0.018$), suggesting that diarrhea is probably caused by other pathogens. No significant difference was found in the other clinical features between the patients with only HMPV detected and the patients with HMPV plus other pathogen(s) detected,

For peer review only - http://bmjopen.bmj.com/site/about/guidelines.xhtml

similar to the results of a previous study.[51]

In our study, RSV (36.8%, 7/19) was the most frequently co-detected pathogen in HMPV-positive patients. Whether HMPV/RSV co-infection is more severe than the respective mono-infections remains unclear. A previous study showed that children with HMPV/RSV co-infection were more likely to develop pneumonia; however, disease severity was not increased.[53] Conversely, Semple et al.[56] reported that HMPV/RSV co-infection can cause more severe bronchiolitis in patients. In our study, no significant difference in the diagnosis of pneumonia was observed between the patients with only HMPV detected and the patients with HMPV plus other pathogen(s) detected. It has been confirmed that a history of prematurity, particular age groups, and the presence of chronic diseases increases the risk of severe LRTI among HMPV- and RSV-infected children.[51] Our study suggests that the clinical manifestations of HMPV infection are complex and diverse; our data will be helpful in the diagnosis of HMPV.

In this study, we analyzed the epidemiological characteristics and clinical characteristics of HMPV in more than 5000 children with ARI in two tertiary hospitals in Guangzhou, which is an international metropolis and the most important political, economic and cultural center in southern China. Therefore, the results are not limited to the two hospital cases, but also represent and reflect HMPV infection in children with ARI in south China and play a positive role in the prevention and diagnosis of HMPV infection in the area.

The study had some limitations. First, collection of data on symptoms and

For peer review only - http://bmjopen.bmj.com/site/about/guidelines.xhtml

281 physical findings in infants and young children requires experienced medical staff, patient's cooperation, and consent from the patient's guardian. Consequently, it is possible we will have incomplete or even an inaccurate description of patients' manifestations. Second, because our study mainly focused on HMPV, other common respiratory pathogens, including bacterial pathogens, were not tested and subsequently our study does not give a fully account of respiratory pathogen infection in hospitalized pediatric patients with ARI. Third, the characteristics of HMPV-negative patients and outcome data (discharge/death) were not analyzed further, because our study only focused on the epidemiological and clinical features of HMPV positive cases. This might affect our understanding of the clinical features of HMPV-positive patients. Despite these shortcomings, our results provide a valuable insight into the epidemiological and clinical characteristics of HMPV present in patients hospitalized with ARI in Guangzhou, southern China.

For peer review only - http://bmjopen.bmj.com/site/about/guidelines.xhtml

Conclusions

HMPV is an important respiratory pathogen in children with ARI in Guangzhou , China, particularly in children under 5 years old. In future, our data can be used by public health authorities and clinicians to improve the management of HMPV infection in children.

For peer review only

For peer review only - http://bmjopen.bmj.com/site/about/guidelines.xhtml

Acknowledgements

We thank the study volunteers for their generous participation. We thank Yinghua Zhou, Haiping Huang, Jing Zhang, and Jing Ma for technical assistance. We thank Professor Xiaofeng Li and Dr. Zhengshi Lin for paper polishing.

Contributors

RZ, WKL, LZ and DLL designed the study. LZ, DLL, WKL, SYQ, DX, XL and LTT performed pathogen testing. DHC and WPT collected the clinical data. All authors participated in the data analysis. LZ, DLL, RZ and WKL drafted the manuscript. All authors read and approved the final version of this manuscript.

Funding

This study was supported by The State Major Infectious Disease Research Program (RZ) (2017ZX10103011-003), National Natural Science Foundation of China (WKL) (31500143), Guangzhou Science and Technology Program key projects (RZ) (201508020252). The funders had no role in study design, data collection and analysis, decision to publish, or preparation of the manuscript.

Competing interests

None declared.

Ethics approval

For peer review only - http://bmjopen.bmj.com/site/about/guidelines.xhtml

The study was approved by The First Affiliated Hospital of Guangzhou Medical University Ethics Committee. Informed written consent was obtained from parents or legal guardians.

Data sharing statement

No additional data are available.

For peer review only

For peer review only - http://bmjopen.bmj.com/site/about/guidelines.xhtml

References

1. van den Hoogen BG, Herfst S, Sprong L, et al. Antigenic and genetic variability of human metapneumoviruses. *Emerg Infect Dis* 2004;10(4):658-66. doi: 10.3201/eid1004.030393

2. van den Hoogen BG, de Jong JC, Groen J, et al. A newly discovered human pneumovirus isolated from young children with respiratory tract disease. *Nat Med* 2001;7(6):719-24. doi: 10.1038/89098

3. Boivin G, Abed Y, Pelletier G, et al. Virological features and clinical manifestations associated with human metapneumovirus: a new paramyxovirus responsible for acute respiratory-tract infections in all age groups. *J Infect Dis* 2002;186(9):1330-4. doi: 10.1086/344319

4. Falsey AR, Walsh EE. Viral pneumonia in older adults. *Clin Infect Dis* 2006;42(4):518-24. doi: 10.1086/499955

5. Kahn JS. Epidemiology of human metapneumovirus. *Clin Microbiol Rev* 2006;19(3):546-57. doi: 10.1128/CMR.00014-06

6. Davis CR, Stockmann C, Pavia AT, et al. Incidence, Morbidity, and Costs of Human Metapneumovirus Infection in Hospitalized Children. *J Pediatric Infect Dis Soc* 2016;5(3):303-11. doi: 10.1093/jpids/piv027

7. Edwards KM, Zhu Y, Griffin MR, et al. Burden of human metapneumovirus infection in young children. *N Engl J Med* 2013;368(7):633-43. doi: 10.1056/NEJMoa1204630

8. Foulongne V, Guyon G, Rodiere M, et al. Human metapneumovirus infection

For peer review only

For peer review only - http://bmjopen.bmj.com/site/about/guidelines.xhtml

in young children hospitalized with respiratory tract disease. *Pediatr Infect Dis J* 2006;25(4):354-9. doi: 10.1097/01.inf.0000207480.55201.f6

9. Heikkinen T, Osterback R, Peltola V, et al. Human metapneumovirus infections in children. *Emerg Infect Dis* 2008;14(1):101-6. doi: 10.3201/eid1401.070251

10. Nicholson KG, McNally T, Silverman M, et al. Rates of hospitalisation for influenza, respiratory syncytial virus and human metapneumovirus among infants and young children. *Vaccine* 2006;24(1):102-8. doi: 10.1016/j.vaccine.2005.02.004

11. Sloots TP, Mackay IM, Bialasiewicz S, et al. Human metapneumovirus, Australia, 2001-2004. *Emerg Infect Dis* 2006;12(8):1263-6. doi: 10.3201/eid1208.051239

12. Williams JV, Edwards KM, Weinberg GA, et al. Population-based incidence of human metapneumovirus infection among hospitalized children. *J Infect Dis* 2010;201(12):1890-8. doi: 10.1086/652782

13. Ebihara T, Endo R, Kikuta H, et al. Seroprevalence of human metapneumovirus in Japan. *J Med Virol* 2003;70(2):281-3. doi: 10.1002/jmv.10391

14. Panda S, Mohakud NK, Pena L, et al. Human metapneumovirus: review of an important respiratory pathogen. *Int J Infect Dis* 2014;25:45-52. doi: 10.1016/j.ijid.2014.03.1394

15. Broor S, Bharaj P, Chahar HS. Human metapneumovirus: a new respiratory

For peer review only - http://bmjopen.bmj.com/site/about/guidelines.xhtml

pathogen. *J Biosci* 2008;33(4):483-93.

16. Jain B, Singh AK, Dangi T, et al. High prevalence of human metapneumovirus subtype B in cases presenting as severe acute respiratory illness: an experience at tertiary care hospital. *Clin Respir J* 2014;8(2):225-33. doi: 10.1111/crj.12064

17. Liu WK, Liu Q, Chen DH, et al. Epidemiology and clinical presentation of the four human parainfluenza virus types. *BMC Infect Dis* 2013;13:28. doi: 10.1186/1471-2334-13-28

18. Liu WK, Chen DH, Liu Q, et al. Detection of human bocavirus from children and adults with acute respiratory tract illness in Guangzhou, southern China. *BMC Infect Dis* 2011;11:345. doi: 10.1186/1471-2334-11-345

19. Liu W, Chen D, Tan W, et al. Epidemiology and Clinical Presentations of Respiratory Syncytial Virus Subgroups A and B Detected with Multiplex Real-Time PCR. *PLoS One* 2016;11(10):e0165108.DOI: 10.1371/journal.pone.08. doi: 10.1371/journal.pone.0165108

20. Williams BG, Gouws E, Boschi-Pinto C, et al. Estimates of world-wide distribution of child deaths from acute respiratory infections. *Lancet Infect Dis* 2002;2(1):25-32.

21. Escobar C, Luchsinger V, de Oliveira DB, et al. Genetic variability of human metapneumovirus isolated from Chilean children, 2003-2004. *J Med Virol* 2009;81(2):340-4. doi: 10.1002/jmv.21399

22. Garcia-Garcia ML, Calvo C, Rey C, et al. Human metapnuemovirus

For peer review only - http://bmjopen.bmj.com/site/about/guidelines.xhtml

infections in hospitalized children and comparison with other respiratory viruses. 2005-2014 prospective study. *PLoS One* 2017;12(3):e0173504.DOI: 10.1371/journal.pone.04. doi: 10.1371/journal.pone.0173504

23. Ljubin-Sternak S, Santak M, Cepin-Bogovic J, et al. Detection of genetic lineages of human metapneumovirus in Croatia during the winter season 2005/2006. *J Med Virol* 2008;80(7):1282-7. doi: 10.1002/jmv.21196

24. McCracken JP, Arvelo W, Ortiz J, et al. Comparative epidemiology of human metapneumovirus- and respiratory syncytial virus-associated hospitalizations in Guatemala. *Influenza Other Respir Viruses* 2014;8(4):414-21. doi: 10.1111/irv.12251

25. Nor'e SS, Sam IC, Mohamad Fakri EF, et al. Phylogenetic analysis of human metapneumovirus among children with acute respiratory infections in Kuala Lumpur, Malaysia. *Trop Biomed* 2014;31(3):562-6.

26. Ordas J, Boga JA, Alvarez-Arguelles M, et al. Role of metapneumovirus in viral respiratory infections in young children. *J Clin Microbiol* 2006;44(8):2739-42. doi: 10.1128/JCM.00164-06

27. Rafiefard F, Yun Z, Orvell C. Epidemiologic characteristics and seasonal distribution of human metapneumovirus infections in five epidemic seasons in Stockholm, Sweden, 2002-2006. *J Med Virol* 2008;80(9):1631-8. doi: 10.1002/jmv.21242

28. Rodriguez PE, Adamo MP, Paglini MG, et al. [Monoinfection of human Metapneumovirus in Cordoba: first clinical and epidemiological research in

For peer review only

For peer review only - http://bmjopen.bmj.com/site/about/guidelines.xhtml

children with respiratory infection in 2011]. *Rev Fac Cien Med Univ Nac Cordoba* 2016;73(3):170-75.

29. Zeng SZ, Xiao NG, Zhong LL, et al. Clinical features of human metapneumovirus genotypes in children with acute lower respiratory tract infection in Changsha, China. *J Med Virol* 2015;87(11):1839-45. doi: 10.1002/jmv.24249

30. Zhu RN, Qian Y, Zhao LQ, et al. Characterization of human metapneumovirus from pediatric patients with acute respiratory infections in a 4-year period in Beijing, China. *Chin Med J (Engl)* 2011;124(11):1623-8.

31. Cai XY, Wang Q, Lin GY, et al. Respiratory virus infections among children in South China. *J Med Virol* 2014;86(7):1249-55. doi: 10.1002/jmv.23931

32. Matsuzaki Y, Itagaki T, Abiko C, et al. Clinical impact of human metapneumovirus genotypes and genotype-specific seroprevalence in Yamagata, Japan. *J Med Virol* 2008;80(6):1084-9. doi: 10.1002/jmv.21194

33. Barbosa Ramirez J, Pulido Dominguez P, Rey Benito G, et al. Human respiratory syncytial virus and metapneumovirus in patients with acute respiratory infection in Colombia, 2000 - 2011. *Rev Panam Salud Publica* 2014;36(2):101-9.

34. Zhang C, Du LN, Zhang ZY, et al. Detection and genetic diversity of human metapneumovirus in hospitalized children with acute respiratory infections in Southwest China. *J Clin Microbiol* 2012;50(8):2714-9. doi: 10.1128/JCM.00809-12

For peer review only - http://bmjopen.bmj.com/site/about/guidelines.xhtml

35. McAdam AJ, Hasenbein ME, Feldman HA, et al. Human metapneumovirus in children tested at a tertiary-care hospital. *J Infect Dis* 2004;190(1):20-6. doi: 10.1086/421120

36. Boivin G, De Serres G, Cote S, et al. Human metapneumovirus infections in hospitalized children. *Emerg Infect Dis* 2003;9(6):634-40. doi: 10.3201/eid0906.030017

37. Maggi F, Pifferi M, Vatteroni M, et al. Human metapneumovirus associated with respiratory tract infections in a 3-year study of nasal swabs from infants in Italy. *J Clin Microbiol* 2003;41(7):2987-91.

38. van den Hoogen BG, van Doornum GJ, Fockens JC, et al. Prevalence and clinical symptoms of human metapneumovirus infection in hospitalized patients. *J Infect Dis* 2003;188(10):1571-7. doi: 10.1086/379200

39. Peiris JS, Tang WH, Chan KH, et al. Children with respiratory disease associated with metapneumovirus in Hong Kong. *Emerg Infect Dis* 2003;9(6):628-33. doi: 10.3201/eid0906.030009

40. Greensill J, McNamara PS, Dove W, et al. Human metapneumovirus in severe respiratory syncytial virus bronchiolitis. *Emerg Infect Dis* 2003;9(3):372-5. doi: 10.3201/eid0903.020289

41. Lin PY, Lin TY, Huang YC, et al. Human metapneumovirus and community-acquired pneumonia in children. *Chang Gung Med J* 2005;28(10):683-8.

42. Ghattas C, Mossad SB. Fatal human metapneumovirus and influenza B virus

For peer review only - http://bmjopen.bmj.com/site/about/guidelines.xhtml

coinfection in an allogeneic hematopoietic stem cell transplant recipient. *Transpl Infect Dis* 2012;14(5):E41-3.DOI: 10.1111/j.399-3062.2012.00766.x. doi: 10.1111/j.1399-3062.2012.00766.x

43. Fathima S, Lee BE, May-Hadford J, et al. Use of an innovative web-based laboratory surveillance platform to analyze mixed infections between human metapneumovirus (hMPV) and other respiratory viruses circulating in Alberta (AB), Canada (2009-2012). *Viruses* 2012;4(11):2754-65. doi: 10.3390/v4112754

44. Kaida A, Kubo H, Goto K, et al. Co-infection of human metapneumovirus with adenovirus or respiratory syncytial virus among children in Japan. *Microbiol Immunol* 2007;51(7):679-83.

45. Pilger DA, Cantarelli VV, Amantea SL, et al. Detection of human bocavirus and human metapneumovirus by real-time PCR from patients with respiratory symptoms in Southern Brazil. *Mem Inst Oswaldo Cruz* 2011;106(1):56-60.

46. Esposito S, Daleno C, Prunotto G, et al. Impact of viral infections in children with community-acquired pneumonia: results of a study of 17 respiratory viruses. *Influenza Other Respir Viruses* 2013;7(1):18-26. doi: 10.1111/j.1750-2659.2012.00340.x

47. Kahn JS. Newly discovered respiratory viruses: significance and implications. *Curr Opin Pharmacol* 2007;7(5):478-83. doi: 10.1016/j.coph.2007.07.004

48. Wolf DG, Greenberg D, Kalkstein D, et al. Comparison of human metapneumovirus, respiratory syncytial virus and influenza A virus lower

For peer review only - http://bmjopen.bmj.com/site/about/guidelines.xhtml

respiratory tract infections in hospitalized young children. *Pediatr Infect Dis J* 2006;25(4):320-4. doi: 10.1097/01.inf.0000207395.80657.cf

49. Wilkesmann A, Schildgen O, Eis-Hubinger AM, et al. Human metapneumovirus infections cause similar symptoms and clinical severity as respiratory syncytial virus infections. *Eur J Pediatr* 2006;165(7):467-75. doi: 10.1007/s00431-006-0105-4

50. Adams O, Weis J, Jasinska K, et al. Comparison of human metapneumovirus, respiratory syncytial virus and Rhinovirus respiratory tract infections in young children admitted to hospital. *J Med Virol* 2015;87(2):275-80. doi: 10.1002/jmv.24025

51. Moe N, Krokstad S, Stenseng IH, et al. Comparing Human Metapneumovirus and Respiratory Syncytial Virus: Viral Co-Detections, Genotypes and Risk Factors for Severe Disease. *PLoS One* 2017;12(1):e0170200.DOI: 10.1371/journal.pone.00. doi: 10.1371/journal.pone.0170200

52. Yan XL, Li YN, Tang YJ, et al. Clinical characteristics and viral load of respiratory syncytial virus and human metapneumovirus in children hospitaled for acute lower respiratory tract infection. *J Med Virol* 2017;89(4):589-97. doi: 10.1002/jmv.24687

53. Caracciolo S, Minini C, Colombrita D, et al. Human metapneumovirus infection in young children hospitalized with acute respiratory tract disease: virologic and clinical features. *Pediatr Infect Dis J* 2008;27(5):406-12. doi: 10.1097/INF.0b013e318162a164

For peer review only - http://bmjopen.bmj.com/site/about/guidelines.xhtml

54. Williams JV, Harris PA, Tollefson SJ, et al. Human metapneumovirus and lower respiratory tract disease in otherwise healthy infants and children. *N Engl J Med* 2004;350(5):443-50. doi: 10.1056/NEJMoa025472

55. Schuster JE, Khuri-Bulos N, Faouri S, et al. Human Metapneumovirus Infection in Jordanian Children: Epidemiology and Risk Factors for Severe Disease. *Pediatr Infect Dis J* 2015;34(12):1335-41. doi: 10.1097/INF.0000000000000892

56. Semple MG, Cowell A, Dove W, et al. Dual infection of infants by human metapneumovirus and human respiratory syncytial virus is strongly associated with severe bronchiolitis. *J Infect Dis* 2005;191(3):382-6. doi: 10.1086/426457

For peer review only

For peer review only - http://bmjopen.bmj.com/site/about/guidelines.xhtml

Figure legends

Figure 1 Age distributions of patients with HMPV.

[a]Date were presented as HMPV positive rate (number of HMPV-positive patients/number of patients in each age group); m: month(s); y: year(s).

Figure 2 Seasonal distribution of HMPV infection in pediatric patients hospitalized with acute respiratory infection from July 2013 to June 2016 in Guangzhou.

For peer review only

Table 1. Distribution of co-pathogens in 19 HMPV-positive patients

Co-pathogens[a]	Patient No.
RSV/HMPV	4
HCoV-OC43/HMPV	3
ADV/HMPV	2
HRV/HMPV	2
MP/HMPV	1
PIV2/HMPV	1
PIV3/HMPV	1
RSV/MP/HMPV	1
RSV/InfA/HMPV	1
RSV/HBoV/HMPV	1
MP/HBoV/HMPV	1
InfA/HBoV/HMPV	1

[a]HMPV-positive patients were tested for 17 common respiratory pathogens.

RSV: respiratory syncytial virus; PIV: parainfluenza virus; InfA: influenza A virus; ADV: adenovirus; HCoV: human coronaviruses; HRV: human rhinovirus; HBoV: human bocavirus; MP: *Mycoplasma pneumoniae.*

Influenza B viruses, parainfluenza virus types 1 and 4, enterovirus, human coronaviruses-HKU1, human coronaviruses-229E, human coronaviruses-NL63, and *Chlamydophila pneumoniae* were not detected.

For peer review only - http://bmjopen.bmj.com/site/about/guidelines.xhtml

Table 2. Clinical presentations of HMPV-positive patients

Diagnosis/Symptom	Total HMPV (n=101)	Patients with only HMPV and HMPV plus other pathogen(s) detected.		
		Single HMPV (n = 83)	Co-pathogens (n= 18)	p value[b]
**Upper respiratory tract infection**				
Nasal obstruction	40(39.6)	34(41.0)	6(33.3)	0.605
Coryza	51(50.5)	44(53.0)	7(38.9)	0.309
Sneeze	1(1.0)	1(1.2)	0(0)	–[c]
Cough	101(100)	83(100)	18(100)	–[d]
Expectoration	78(77.2)	66(79.5)	12(66.7)	0.351
Hoarseness	1(1.0)	1(1.2)	0(0)	–[c]
**Lower respiratory tract infection**				
Abnormal pulmonary breath sound [a]	92(91.1)	75(90.4)	17(94.4)	0.582
Wheezing	47(46.5)	40(48.2)	7(38.9)	0.473
Anhelation	24(23.8)	21(25.3)	3(16.7)	0.551
Increasing lung markings[e]	6(7.1)	4(5.8)	2(13.3)	0.304
Bronchiolitis[e]	8(9.5)	7(10.1)	1(6.7)	0.677
Pneumonia[e]	11(13.1)	10(14.5)	1(6.7)	0.415
Bronchopneumonia[e]	56(66.7)	46(66.7)	10(66.7)	0.626
**Systemic influenza-like symptoms**				
Fever (≥38˚C)	89(88.1)	76(91.6)	13(72.2)	**0.037**
Chill	7(6.9)	6(7.2)	1(5.6)	0.8
Debilitation	11(10.9)	11(13.3)	0(0)	–[c]
**Gastrointestinal illness**				
Vomiting	21(20.8)	16(19.3)	5(27.8)	0.522
Poor appetite	19(18.8)	17(20.5)	2(11.1)	0.513
Diarrhea	7(7.0)	3(3.6)	4(22.2)	**0.018**

Data are presented as No. (%) of each group.

Percentages sum to >100% because some patients had >1 clinical presentations.

[a]Including phlegmatic, wheezing, bubbling, and moist rales.

[b]Two-tailed χ^2 test comparing the distribution of each illness or diagnosis between patients with only HMPV and HMPV plus other pathogen(s) detected.

[c]Not tested because the number of positive samples obtained was too small.

[d]Not tested because the prevalence was 100%.

[e]Diagnosed by chest radiography, and a total of 84 HMPV-positive patients (69

For peer review only - http://bmjopen.bmj.com/site/about/guidelines.xhtml

542 patients with only HMPV detected and 15 patients with HMPV plus other pathogen(s) detected) were examined.

For peer review only - http://bmjopen.bmj.com/site/about/guidelines.xhtml

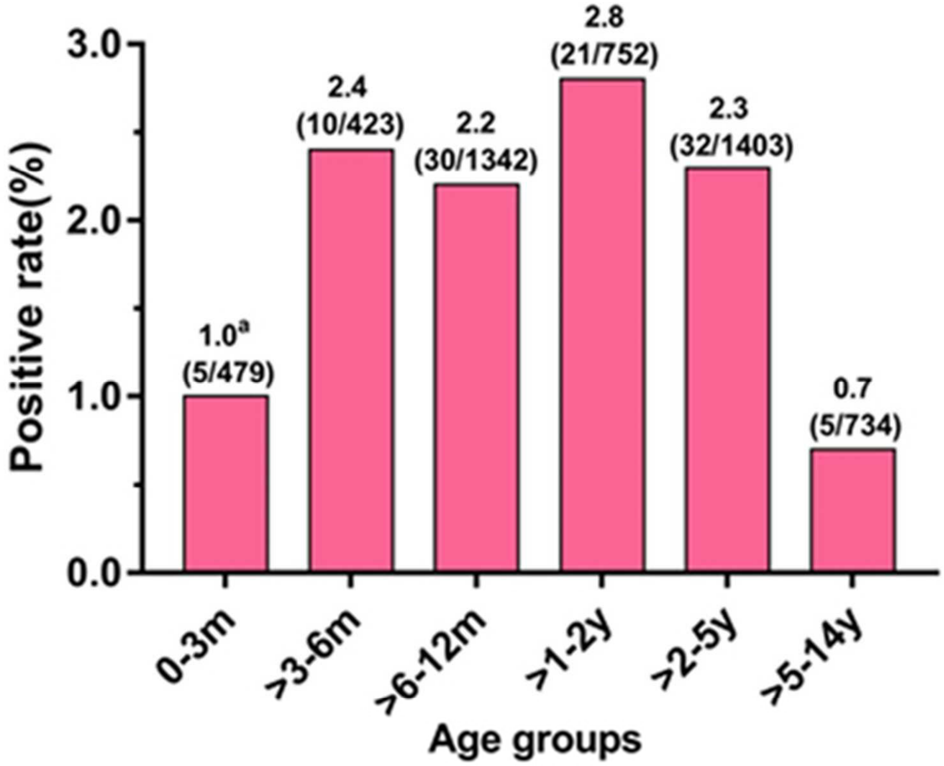

Figure 1 Age distributions of patients with HMPV.
aDate were presented as HMPV positive rate (number of HMPV-positive patients/number of patients in each age group); m: month(s); y: year(s).

42x35mm (300 x 300 DPI)

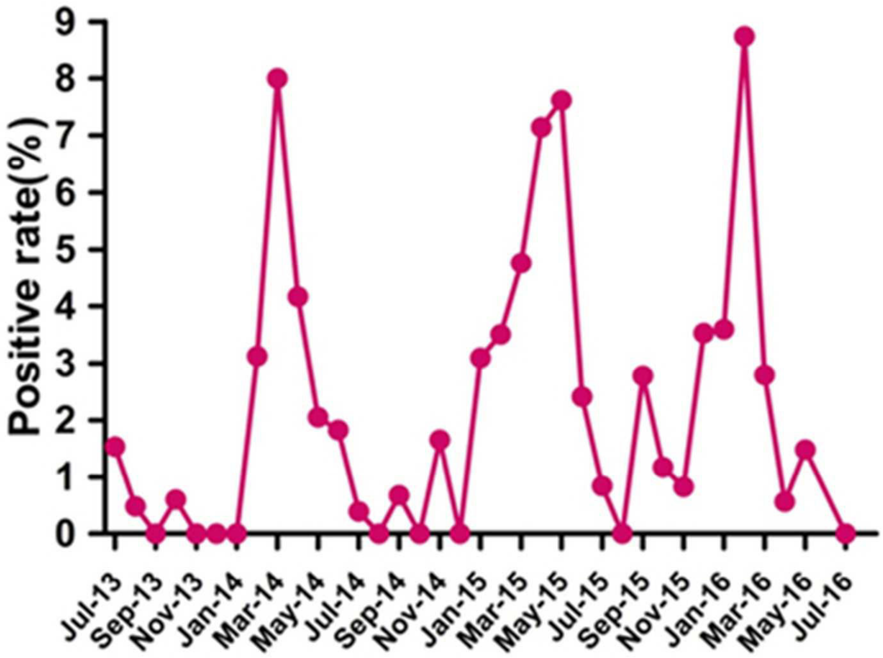

Figure 2 Seasonal distribution of HMPV infection in pediatric patients hospitalized with acute respiratory infection from July 2013 to June 2016 in Guangzhou.

39x31mm (300 x 300 DPI)

STROBE 2007 (v4)Statement—Checklist of items that should be included in reports of *cross-sectional studies*

Section/Topic	Item #	Recommendation	Reported on page #
**Title and abstract**	1	(*a*) Indicate the study's design with a commonly used term in the title or the abstract	1
		(*b*) Provide in the abstract an informative and balanced summary of what was done and what was found	2-3
**Introduction**			
Background/rationale	2	Explain the scientific background and rationale for the investigation being reported	4
Objectives	3	State specific objectives, including any prespecified hypotheses	4-5
**Methods**			
Study design	4	Present key elements of study design early in the paper	6
Setting	5	Describe the setting, locations, and relevant dates, including periods of recruitment, exposure, follow-up, and data collection	6
Participants	6	(*a*) Give the eligibility criteria, and the sources and methods of selection of participants	6
Variables	7	Clearly define all outcomes, exposures, predictors, potential confounders, and effect modifiers. Give diagnostic criteria, if applicable	6-7
Data sources/ measurement	8*	For each variable of interest, give sources of data and details of methods of assessment (measurement). Describe comparability of assessment methods if there is more than one group	7
Bias	9	Describe any efforts to address potential sources of bias	*6*
Study size	10	Explain how the study size was arrived at	6
Quantitative variables	11	Explain how quantitative variables were handled in the analyses. If applicable, describe which groupings were chosen and why	7-8
Statistical methods	12	(*a*) Describe all statistical methods, including those used to control for confounding	8
		(*b*) Describe any methods used to examine subgroups and interactions	8
		(*c*) Explain how missing data were addressed	7
		(*d*) If applicable, describe analytical methods taking account of sampling strategy	8
		(*e*) Describe any sensitivity analyses	
**Results**			

For peer review only

Participants	13*	(a) Report numbers of individuals at each stage of study—eg numbers potentially eligible, examined for eligibility, confirmed eligible, included in the study, completing follow-up, and analysed	9
		(b) Give reasons for non-participation at each stage	10
		(c) Consider use of a flow diagram	
Descriptive data	14*	(a) Give characteristics of study participants (eg demographic, clinical, social) and information on exposures and potential confounders	9-11
		(b) Indicate number of participants with missing data for each variable of interest	9
Outcome data	15*	Report numbers of outcome events or summary measures	9-11
Main results	16	(*a*) Give unadjusted estimates and, if applicable, confounder-adjusted estimates and their precision (eg, 95% confidence interval). Make clear which confounders were adjusted for and why they were included	9-11
		(*b*) Report category boundaries when continuous variables were categorized	9-10
		(*c*) If relevant, consider translating estimates of relative risk into absolute risk for a meaningful time period	
Other analyses	17	Report other analyses done—eg analyses of subgroups and interactions, and sensitivity analyses	
**Discussion**			
Key results	18	Summarise key results with reference to study objectives	12-15
Limitations	19	Discuss limitations of the study, taking into account sources of potential bias or imprecision. Discuss both direction and magnitude of any potential bias	15-16
Interpretation	20	Give a cautious overall interpretation of results considering objectives, limitations, multiplicity of analyses, results from similar studies, and other relevant evidence	12-16
Generalisability	21	Discuss the generalisability (external validity) of the study results	12-16
**Other information**			
Funding	22	Give the source of funding and the role of the funders for the present study and, if applicable, for the original study on which the present article is based	18

*Give information separately for cases and controls in case-control studies and, if applicable, for exposed and unexposed groups in cohort and cross-sectional studies.

Note: An Explanation and Elaboration article discusses each checklist item and gives methodological background and published examples of transparent reporting. The STROBE checklist is best used in conjunction with this article (freely available on the Web sites of PLoS Medicine at http://www.plosmedicine.org/, Annals of Internal Medicine at http://www.annals.org/, and Epidemiology at http://www.epidem.com/). Information on the STROBE Initiative is available at www.strobe-statement.org.

For peer review only
